# Controlling the Superconductivity of Nb_2_Pd_*x*_S_5_ via Reversible Li Intercalation

**DOI:** 10.1021/acs.inorgchem.3c03524

**Published:** 2024-01-04

**Authors:** Mahmoud Elgaml, Sunita Dey, Jiayi Cen, Maxim Avdeev, David O. Scanlon, Clare P. Grey, Simon J. Clarke

**Affiliations:** † Department of Chemistry, University of Oxford, Inorganic Chemistry Laboratory, South Parks Road, Oxford OX1 3QR, U.K.; ‡ Department of Chemistry, University of Cambridge, Lensfield Road, Cambridge CB2 1EW, U.K.; § Department of Chemistry, University College London, 20 Gordon Street, London WC1H 0AJ, U.K.; ∥ Australian Nuclear Science and Technology Organisation, New Illawarra Road, Lucas Heights, NSW 2234, Australia; ∇ School of Chemistry, The University of Sydney, Sydney 2006, Australia

## Abstract

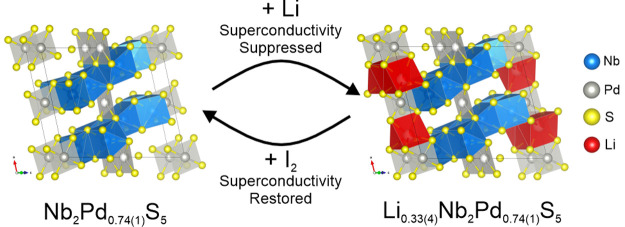

The Nb_2_Pd_*x*_S_5_ (*x* ≈ 0.74) superconductor with a *T*_c_ of 6.5 K is reduced by the intercalation of lithium in ammonia solution or electrochemically to produce an intercalated phase with expanded lattice parameters. The structure expands by 2% in volume and maintains the *C*2/*m* symmetry and rigidity due to the PdS_4_ units linking the layers. Experimental and computational analysis of the chemically synthesized bulk sample shows that Li occupies triangular prismatic sites between the layers with an occupancy of 0.33(4). This level of intercalation suppresses the superconductivity, with the injection of electrons into the metallic system observed to also reduce the Pauli paramagnetism by ∼40% as the bands are filled to a Fermi level with a lower density of states than in the host material. Deintercalation using iodine partially restores the superconductivity, albeit at a lower *T*_c_ of ∼5.5 K and with a smaller volume fraction than in fresh Nb_2_Pd_*x*_S_5_. Electrochemical intercalation reproduces the chemical intercalation product at low Li content (<0.4) and also enables greater reduction, but at higher Li contents (≥0.4) accessed by this route, phase separation occurs with the indication that Li occupies another site.

## Abstract 2

The superconductor Nb_2_Pd_0.74_S_5_ (*T*_c_ ≈ 6.5 K) is intercalated with lithium both chemically and electrochemically. The intercalate has expanded lattice parameters with the *C*2/*m* symmetry maintained. At low Li contents, Li occupies triangular prismatic sites between the layers. Upon intercalation, the superconductivity is supressed but can be partially restored upon deintercalation with iodine. At higher Li content, phase separation occurs.

## 1 Introduction

A majority of transition-metal chalcogenides that have been well studied are binary compounds. However, with the improvement in air-sensitive synthesis and characterization methods, there has been an increase in the investigations of the chemical and physical properties of ternary chalcogenides. Research in these compounds has become more prominent recently, with phenomena such as the highly anisotropic ferromagnetism observed in Fe_3_GeTe_2_ showing potential for applications in spintronics.^1−3^ Chalcogenide perovskites, for example, BaZrS_3_, have been found to compete with the well-known hybrid lead halide perovskites in photovoltaics with greater stability and reduced toxicity.^4,5^ Ternary chalcogenide compounds exhibit a variety of different structure types, from the highly two-dimensional, such as the excitonic insulator candidate Ta_2_NiSe_5_,^6^ to layers with interlayer connectivity, such as in Ta_2_PdS_5_,^7^ to the zigzag arrangement of planes in Nb_2_Pd_3_Se_8_.^8^

Because of the high polarizability of sulfide and the heavier chalcogenide ions compared with oxide anions,^9^ layered structures are common and over two-thirds of the binary metal chalcogenides are layered. The intercalation chemistry of these compounds has been extensively investigated, most notably in the area of battery materials, such as the prototypical secondary battery positive electrode system involving reversible intercalation of Li into TiS_2_.^10^ These topochemical reactions have been widely studied with the aim of tuning their properties; for example, Li/NH_3_ intercalation into the layered polymorph of FeSe increased the superconducting transition temperature (*T*_c_) significantly from 8.5 to 43 K.^11^ Deintercalation of K from the thermodynamically stable KCo_2_Se_2_ can form metastable layered CoSe, with the Curie temperature of the ferromagnetic transition decreasing from ∼82 to 10 K.^12^

For ternary chalcogenides, intercalation chemistry is less well explored. However, the layered members of this class have the potential for intercalation chemistry to tune the electron count, introduce mixed valence, and potentially bring forth more exotic properties, as seen in the ethylenediamine intercalation of Ta_2_PdSe_6_ reported to induce superconductivity with *T*_c_ = 4.5 K.^13^

**Figure 1:**
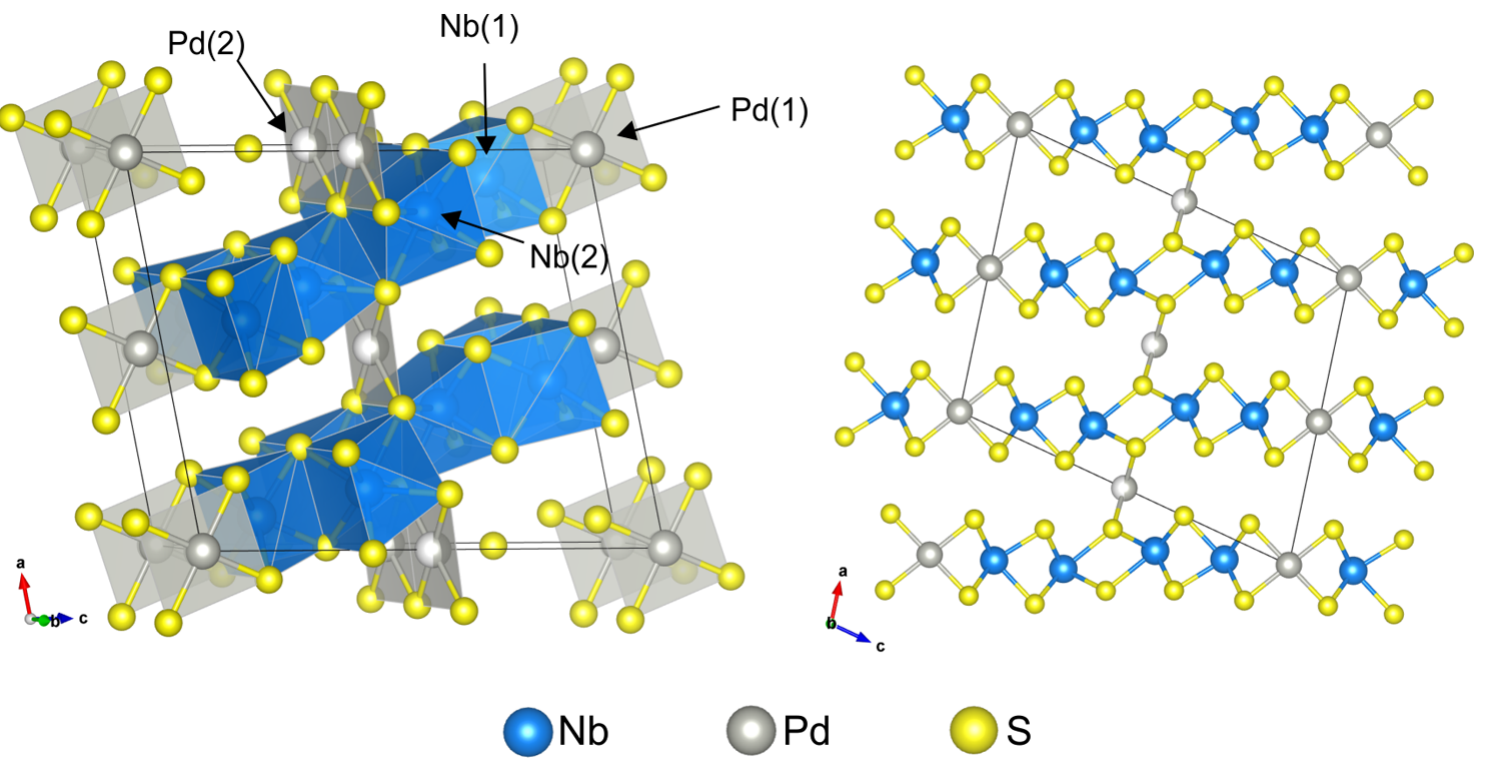
Structure of Nb_2_Pd_*x*_S_5_ showing the layered arrangement with Pd linkages. The different metal coordination sites are also indicated.

Nb_2_Pd_*x*_S_5_ is a member of the M_2_Pd_*x*_Ch_5_ (M = Nb, Ta; Ch = S, Se) superconductor family. First reported by Zhang et al.,^14^ Nb_2_Pd_*x*_S_5_, depicted in Figure 1 is monoclinic with space group *C*2/*m* and consists of NbS_6_ triangular prisms [Nb(1)], NbS_7_ monocapped triangular prisms [Nb(2)], and PdS_4_ square planar [Pd(1)] units arranged in layers. The layers are linked by additional PdS_4_ [Pd(2)] units. This site in the linkage is intrinsically deficient in Pd, with the site approximately half occupied. A sample with the reported composition of Nb_2_Pd_0.81_S_5_ was observed to have a superconducting transition temperature (*T*_c_) of 6.6 K.^14^ Although *T*_c_ is relatively low, this compound was reported to have a remarkably large and anisotropic upper critical field, *H*_c__2_, of up to 37 T,^14^ violating by a factor of 3, of the Pauli paramagnetic limit, which describes the maximum possible field strength (*H*_c__2_ ≈ 1.85*T*_c_ ≈ 12 T) for a Bardeen–Cooper–Schrieffer (BCS) superconductor.^15^

Research on these compounds has focused on the modification of the superconductivity. This has been mainly achieved by substitutional hole or electron doping by making substitutions at the Pd site. Shen et al. reported Nb_2_(Pd_1–*y*_R_*y*_)_0.76_S_5_ (R = Ir, Ag).^16^ It was found that superconductivity can be enhanced by partial substitution of Pd by Ir with a maximum *T*_c_ of ∼8 K when *y* = 0.4, but upon further hole-doping, *T*_c_ is suppressed. With electron doping, with Ag replacing Pd, *T*_c_ decreases until there is complete suppression when 40% of the Pd is replaced by Ag. Superconductivity is also completely suppressed when 15% of the Pd is replaced by Ni in an isoelectronic substitution.^17^ By replacing sulfide in Nb_2_PdS_5_ with selenide, *T*_c_ decreases with increasing Se content with complete suppression at 50% Se substitution.^18^

No intercalation chemistry has been reported for this compound; however, the van der Waals gap between the layers makes this material a promising candidate for the intercalation of small ions. Even though the layers are linked by the interlayer Pd ions, as shown in Figure 1, there are large channels in the structure. In this report, the Li intercalation via chemical and electrochemical methods into Nb_2_Pd_*x*_S_5_ is shown to completely suppress the superconductivity, with deintercalation partially restoring it. A detailed structural study is performed by using a combination of X-ray and neutron powder diffraction, solid-state nuclear magnetic resonance (NMR) spectroscopy, and computation.

## 2 Experimental Section

### 2.1 Synthesis

Due to the air sensitivity of the intercalated products, all treatment and handling of materials were carried out in an argon-filled dry glovebox (H_2_O, O_2_ contents < 1 ppm) or using a Schlenk line. Polycrystalline samples of Nb_2_Pd_*x*_S_5_ were synthesized by a direct combination of elements. A 2:0.85:6 ratio of Nb:Pd:S was ground together, pelletized, sealed in a dried evacuated silica ampule, heated to 850 °C at a rate of 1 °C/min and held for 24 h, and then left to cool at the natural rate of the furnace.

#### 2.1.1 Chemical Intercalation

Intercalation of Li into Nb_2_Pd_*x*_S_5_ was carried out using Li/ammonia solution on a Schlenk line. Attempts to use other chemical lithiating agents produced multiphase products. The Schlenk tube containing approximately 1 g of the host Nb_2_Pd_*x*_S_5_ and 0.6–1.0 mol equiv of Li (see below) was evacuated on the Schlenk line and cooled to −78 °C using a solid CO_2_/propan-2-ol cooling bath. Approximately 10 cm^3^ of ammonia was condensed into the tube via the vacuum line. The solution was left to stir for up to 3 h and warmed to room temperature to enable the ammonia to evaporate off before final evacuation and removal to the dry box. (Safety note: ammonia is volatile and highly toxic. At all times, venting of the system was available via a mercury manometer attached to the Schlenk line located in a fume hood.)

#### 2.1.2 Chemical Deintercalation

Deintercalation of the intercalated material was carried out using iodine as the deintercalant. Three moles of I_2_ per mole of the Li-intercalated Nb_2_Pd_*x*_S_5_ phase (i.e., a large excess) was placed in a Schlenk tube and exposed to a nitrogen atmosphere. Approximately 10 cm^3^ of dry acetonitrile was used to fully dissolve the iodine, forming a dark-red solution. The whole solution was then transferred to another Schlenk tube containing the intercalated sample and left to stir for 3 days. The suspension was filtered and washed three times using dry acetonitrile and left to dry for 1 h under a dynamic vacuum.

#### 2.1.3 Electrochemical Intercalation

Electrochemical Li intercalation was conducted using coin cells (CR2032, Cambridge Energy Solutions) assembled in an Ar-filled glovebox. The Nb_2_Pd_*x*_S_5_ phase was mixed with Super P Carbon (Timcal) and polyvinylidene fluoride (PVDF) binder (weight ratio 80:10:10) to prepare the cathode. A coin cell was assembled with the cathode, a borosilicate glass fiber separator (Whatman, 15 mm diameter) soaked with 75 μL of electrolyte, and a Li counter electrode (diameter 13 mm). LiPF_6_ (1 M) in ethylene carbonate (EC):dimethyl carbonate (DMC) (1:1) was used as the electrolyte. Galvanostatic (dis)charge was carried out at room temperature with a Lanhe battery cycler (Wuhan Land Electronics Co. Ltd.) at rate *C*/10, where *C* is defined as the theoretical capacity of the compound (the theoretical capacity of the composition that we deduced for the host phase Nb_2_Pd_0.74_S_5_ (see below) is ∼63 mAh/g for 1 mol of Li^+^ intercalation per formula unit) and *C*/10 means fully charging or discharging over 10 h. Prior to *ex situ* measurements, batteries were disassembled inside the Ar-filled glovebox, and the electrode mixture was rinsed three times with DMC and dried in the glovebox antechamber (under vacuum) for 30 min.

### 2.2 Structural Characterization

#### 2.2.1 X-ray and Neutron Diffraction Measurements

Detailed structural information for the NbPd_*x*_S_5_ host and for the intercalated products was obtained by synchrotron powder X-ray diffraction (PXRD). The data was collected on the I11 beamline at the Diamond Light Source, Harwell, United Kingdom.^19^ The synchrotron X-rays were monochromated to have a wavelength of approximately 0.825 Å, which was measured accurately at the start of each session of beam time using a silicon standard. Samples were prepared by grinding the material with an equal volume of amorphous silica glass to limit the absorption and preferred orientation and packed into flame-sealed 0.5-mm-diameter borosilicate capillaries.

Preliminary PXRD patterns were measured using a Bruker D8 Advance Eco X-ray diffractometer (Bragg–Brentano geometry, θ–2θ) operated at 40 kV and 25 mA with Cu Kα radiation. For these laboratory PXRD measurements, the powder samples were sprinkled on a borosilicate glass coverslip using a minimal amount of Dow Corning high-vacuum silicone grease as an adhesive. These were mounted in an aluminum gastight sample holder under an inert atmosphere.

Powder neutron diffraction (PND) was carried out on the Echidna instrument at the Australian Nuclear Science and Technology Organisation (ANSTO), Australia.^20^ The instrument uses the OPAL nuclear reactor as the neutron source. Neutron diffraction was measured using a wavelength of 1.622 Å by using a Ge (*hkl* = 335) monochromator. The samples were contained in 6-mm-diameter vanadium cans sealed with indium gaskets. Diffraction patterns were analyzed using Rietveld refinement, which was performed using the Topas Academic Version 6 software.^21^

#### 2.2.2 Magnetometry

Magnetic susceptibility measurements were conducted using a Quantum Design MPMS-3 Superconducting Quantum Interference Device (SQUID) magnetometer. Gelatin capsules were used to contain accurately weighed powder samples of about 20 mg in mass. To test for superconductivity, measurements were performed on warming in a direct current (DC) field of 10 Oe in the temperature range of 1.8–8 K after first cooling in zero applied field (zero-field cooling: ZFC) and then after cooling in the applied field of 10 Oe (field cooling: FC). To determine the magnetic susceptibility, the magnetic moment of the sample was measured as a function of temperature at fields of 4 and 3 T, above the saturation fields of about 1 T of possible small amounts of ferromagnetic impurity. The bulk susceptibility was determined from the difference between the two high-field measurements made at each temperature.

#### 2.2.3 Solid-State NMR Spectroscopy

^7^Li magic-angle spinning (MAS) NMR experiments were performed with a Bruker Avance 300 MHz (7.05 T) spectrometer (Larmor frequency for ^7^Li of 116.6 MHz) employing a 2.5 mm Bruker probe. Samples were packed into a 2.5 mm ZrO_2_ rotor inside the glovebox and spun at speeds of between 25 and 35 kHz. A rotor-synchronized Hahn echo sequence (90°-τ-180°-τ-acquisition) with a π/2 pulse length of 1.0 μs and a recycle delay of 0.1 s was used. The spectra were referenced to an external LiF standard at −1.0 ppm. The Bruker Topspin software (version 4.0.7) was used for raw data processing.

#### 2.2.4 Computational Analysis

The Vienna Ab Initio Simulation Package (VASP) (version 5.4.4), a plane wave density functional theory (DFT) code, was used to calculate the electronic densities of states (DOS) and electronic band structure and to determine Li site energies.^22−24^ A plane wave cutoff energy of 600 eV and Γ-centered Monkhorst-Pack grids with a maximum spacing of 0.043 2π Å^–1^ were used with projector-augmented-wave pseudopotentials from data set PBE.54 (Li_sv, Nb_pv, Pd, and S).^25,26^ The PBEsol generalized gradient approximation (GGA) functional was used to model the exchange and correlation effects.^27^ Tolerances of 10^–6^ eV and 10^–2^ eV Å^–1^ were applied to the total energy and forces during electronic minimization and geometry optimization, respectively.

The DOS were computed using denser *k*-point grids with a maximum spacing of 0.025 2π Å^–1^. Analysis of the DOS was conducted using the sumo python package.^28^ For Li site-energy calculations, input files were prepared using the doped python package with a 32-atom supercell.^29,30^ The force tolerance was increased to 2 × 10^–2^ eV Å^–1^ for Li interstitials during relaxations, with fixed lattice parameters. The pymatgen and ase python packages were used to manipulate structures and to assist symmetry analysis of the Li sites.^31,32^

## 3 Results and Discussion

### 3.1 Characterization of the Nb_2_Pd_0.74_S_5_ Intercalation Host

In the synthesis of the parent material Nb_2_Pd_*x*_S_5_, a 20% excess of S (3 moles per mole of Nb rather than 2.5) was used, as reported by Zhang et al.^14^ Using stoichiometric amounts of S led to the formation of binary Nb-S side phases. A substoichiometric amount of Pd was found to be optimal for the synthesis. Using a Pd stoichiometry corresponding to *x* = 1 in the formula Nb_2_Pd_*x*_S_5_ produced a PdS side phase at ∼5% by mass. Lowering the Pd content to *x* = 0.85 in Nb_2_Pd_*x*_S_5_ reduced the PdS impurity to <3% by mass. For the samples reported here, with a starting stoichiometry of *x* = 0.85 in Nb_2_Pd_*x*_S_5_, the site occupancy of Pd, the strongest X-ray scatterer in the system, was refined to be 0.74(1) with the independent and PXRD and PND measurements giving consistent values. The side phase PdS (<3% by mass) accounted for the additional Pd and S used in the synthesis. Attempts to target exactly the refined composition led to less pure products. Structural parameters from the Rietveld refinements are provided in Table 1. SEM imaging shows a homogeneous distribution of the elements with EDX mapping on five points on the crystallite (Figure S1), giving an average stoichiometry of Nb_2_Pd_0.78(1)_S_4.9(1)_, also in line with the Rietveld refinements. We refer to the compound in what follows below as Nb_2_Pd_0.74_S_5_. The compound has a *T*_c_ of 6.5 K and a superconducting volume fraction of ∼80% (Figure 3). Varying the starting Pd content had no significant effect on the superconducting properties. Yu et al. reported the synthesis of Nb_2_Pd_*x*_S_5−δ_ (0.6 < *x* < 1; 0 < δ < 0.62) single-crystal fibers with *T*_c_ ranging from 4.67 to 7.43 K, but no reliable trend in the *T*_c_ with the reported composition was observed.^33^ In contrast, the selenide analogue was reported to have a positive correlation between Pd occupancy and *T*_c_, with the latter ranging from 3.4 to 5.5 K for reported compositions 0.8 < *x* < 1.3.^34^

**Figure 2:**
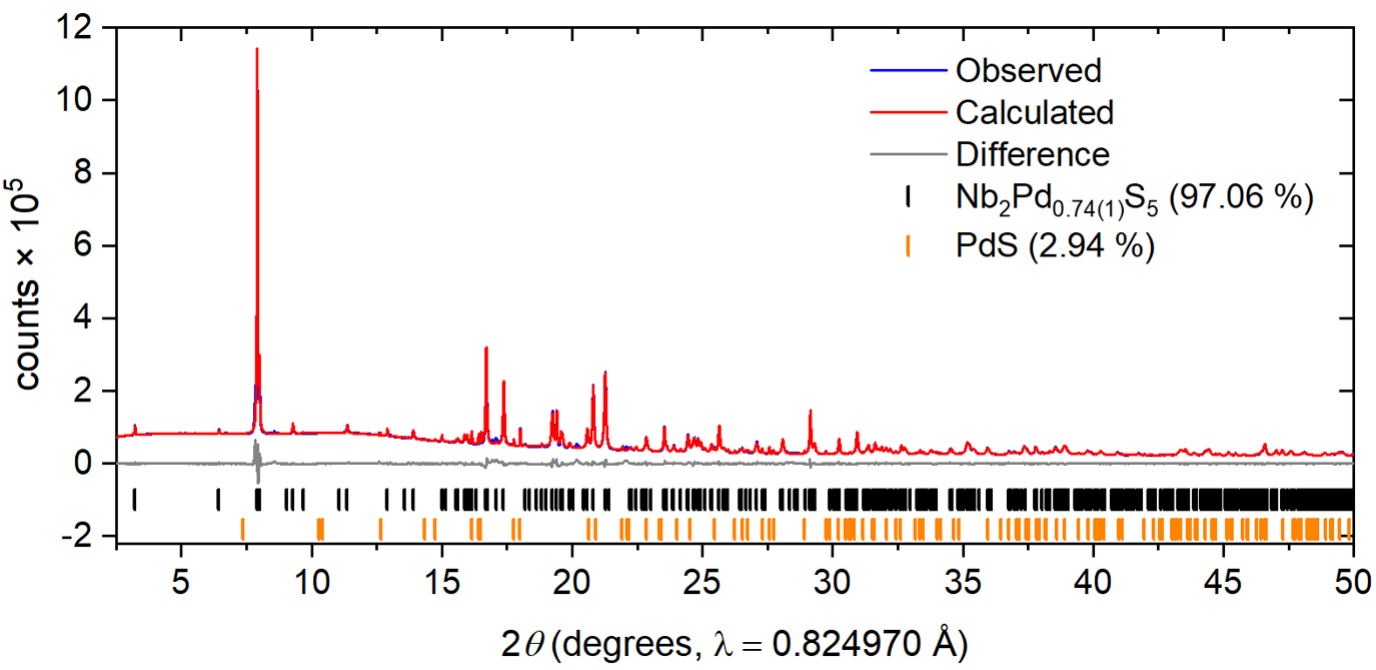
Rietveld refinement of Nb_2_Pd_0.74(1)_S_5_ against synchrotron X-ray powder diffraction data at ambient temperature. *R*_wp_: 3.73%. The blue and red lines show the observed and calculated diffraction patterns, with the gray line being the difference between them. The tick marks show the Bragg reflections for the phases with weight percentages from the refinements given.

**Figure 3:**
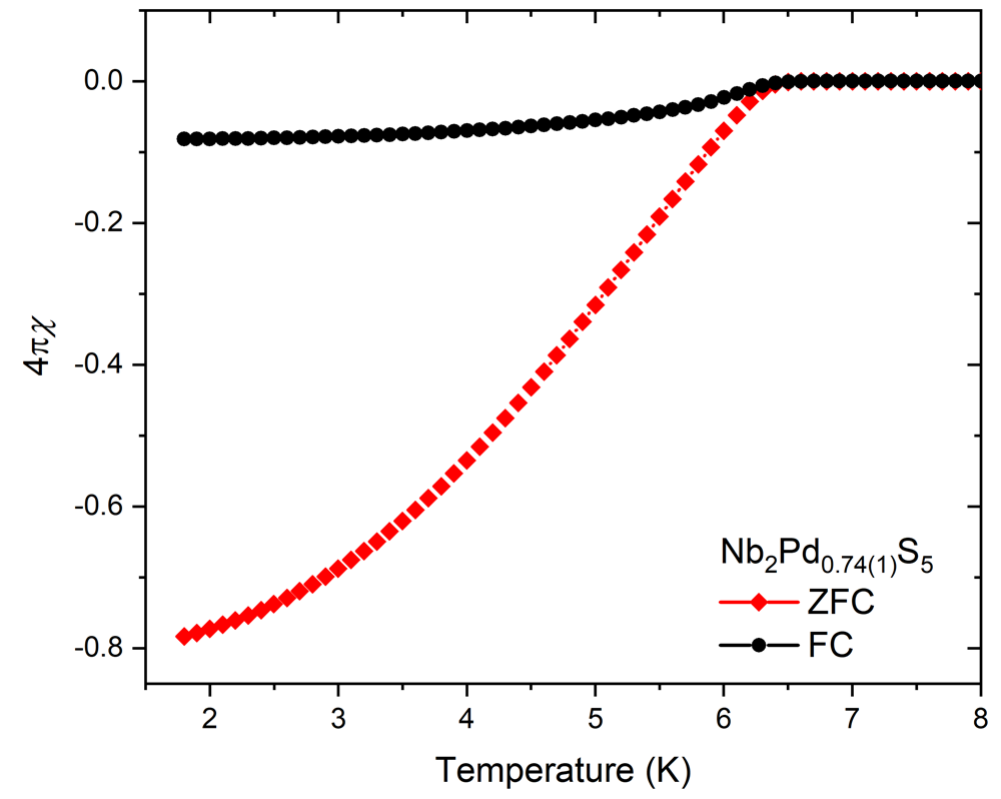
Superconducting volume fraction vs temperature plot showing zero-field-cooled and field curves measured at 10 Oe.

**Table 1:** Structural Parameters of Nb_2_Pd_0.74_S_5_, the Intercalated Phases, and the Deintercalated Phase

**Compound**	**Nb**_**2**_**Pd**_**0.74(1)**_**S**_**5**_	**Li**_**x**_**Nb**_**2**_**Pd**_**0.741)**_**S**_**5**_	**Li**_**0.33(4)**_**Nb**_**2**_**Pd**_**0.74(1)**_**S**_**5**_	**Nb**_**2**_**Pd**_**0.74(1)**_**S**_**5**_
	Parent	Intercalate	Intercalate	Deintercalate
**Instrument**	I11 Beamline	I11 Beamline	Echidna	I11 Beamline
***a*****/Å**	12.1448(1)	12.3390(5)	12.3428(12)	12.1892(2)
***b*****/Å**	3.27971(2)	3.2933(1)	3.2914(2)	3.28279(3)
***c*****/Å**	15.0798(1)	15.3447(6)	15.3390(15)	15.1410(2)
**Volume/Å**^**3**^	585.04(1)	598.03(4)	597.5(1)	588.10(2)
**β/deg**	103.161(9)	106.444(1)	106.489(7)	103.906(2)
**Mean Bond Lengths/Å**				
**Nb(1)–S**	2.469(3)	2.463(3)	2.433(7)	2.472(3)
**Nb(2)–S**	2.523(3)	2.545(3)	2.528(7)	2.539(4)
**Pd(1)–S**	2.358(5)	2.373(6)	2.286(11)	2.348(6)
**Pd(2)–S**	2.403(5)	2.390(6)	2.401(11)	2.408(5)

**Figure 4:**
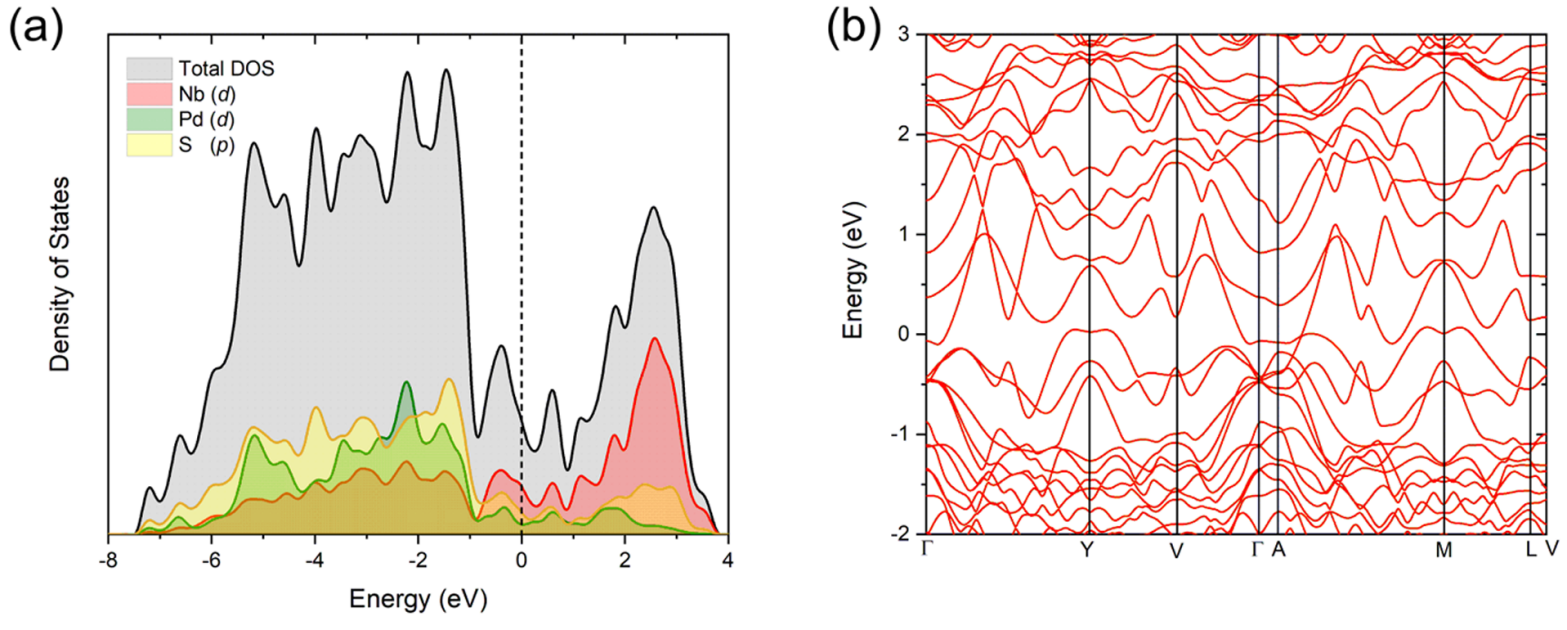
(a) Electronic density of states calculated for a stoichiometry of Nb_2_PdS_5_ and (b) electronic band structure showing the metallic nature and the hybridization of the bands.

The calculated electronic DOS (Figure 4(a)) and band structure (Figure 4(b)) confirm the metallic nature with no gap at the Fermi level (*E*_F_), consistent with previous reports.^14,35^ DFT calculations were performed on the stoichiometric Nb_2_PdS_5_ without the Pd deficiency to avoid the construction of the computationally unwieldy supercells that would be required for Pd-deficient systems in the absence of Pd/vacancy ordering. The change in Pd(2) site occupancy is not expected to modify the topology of the band structure but may cause a slight shift in the Fermi level (defined as Energy = 0 in Figure 4). Stoichiometric Nb_2_PdS_5_, although electron-rich relative to the experimental composition, was deemed sufficient to display the important features of the electronic structure. The valence band (where Energy < 0) is dominated by strongly hybridized Pd 4d and S 3p states. Around the Fermi level, the bands mainly consist of Nb 4d and S 3p hybridized states, which also form the majority of the conduction band.

### 3.2 Structure Refinement of the Li Intercalate of Nb_2_Pd_0.74_S_5_

**Figure 5:**
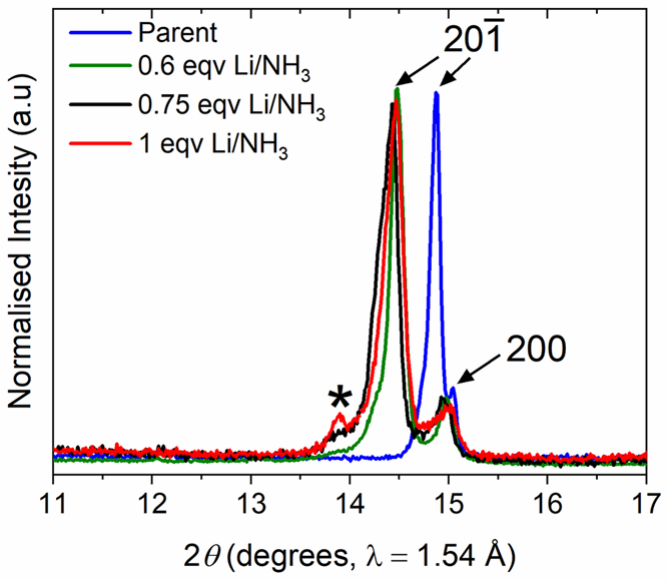
X-ray diffraction pattern of the 201 and 200 Bragg reflections with varying starting Li stoichiometries. The main 201 Bragg reflection can be seen to shift to lower angle upon intercalation. The asterisk indicates a peak appearing at higher Li content possibly due to a side phase forming.

Nb_2_Pd_0.74_S_5_ was first intercalated by reacting lithium dissolved in ammonia with the sulfide with a 1:1 molar ratio of Li:Nb_2_Pd_0.74_S_5_. The powder remained black in color, with the dark-blue color of the solvated electrons decolorizing in minutes. This produced an intercalated phase with an expanded unit cell according to PXRD. However, as seen in Figure 5, a side phase appears to form with a small side peak at a slightly lower angle than the shifted main peak (201 Bragg reflection). This peak diminishes in intensity when less Li is used and was not significant when a 0.6:1 Li:Nb_2_Pd_0.74_S_5_ ratio was used.

**Figure 6:**
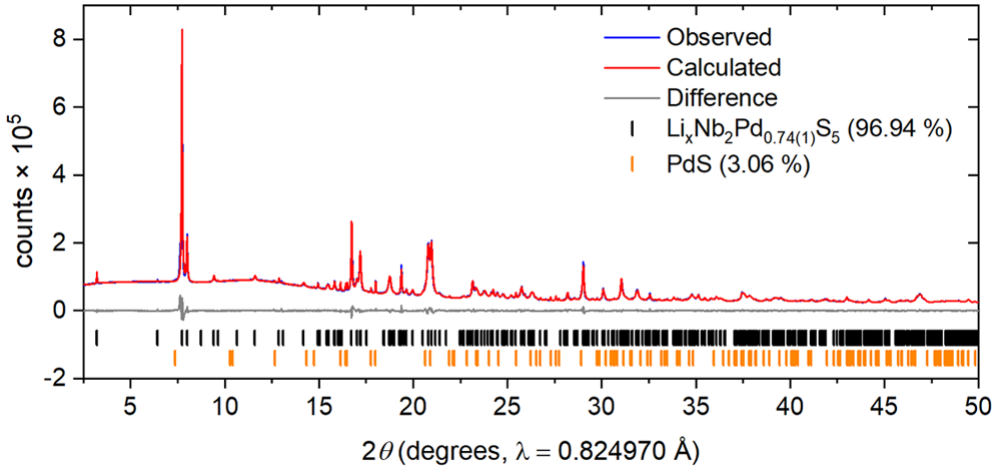
Rietveld refinement of the synchrotron X-ray diffraction pattern of Li_*x*_Nb_2_Pd_0.74(1)_S_5_. *R*_wp_: 2.79%. The Li content here is labeled as *x* as the Li content cannot be refined from the X-ray data, although it is refined in the neutron diffraction data.

The diffraction pattern of the intercalated phase (using 0.6 mol equiv of Li) was measured on the I11 beamline, Diamond Light Source, with the Rietveld refinement (Figure 6) determining that the unit cell expanded by approximately 0.2, 0.01, and 0.26 Å in the *a*, *b*, and *c* directions, respectively, with an increase of ∼3.3° in the monoclinic angle and the *C*2/*m* space group being maintained. Comparing the mean bond lengths shown in Table 1, which are determined with much lower precision than the lattice parameters, there appears to be no significant change (the distances barely differ at the 3σ level), reflecting the small increase in unit cell dimensions.

**Figure 7:**
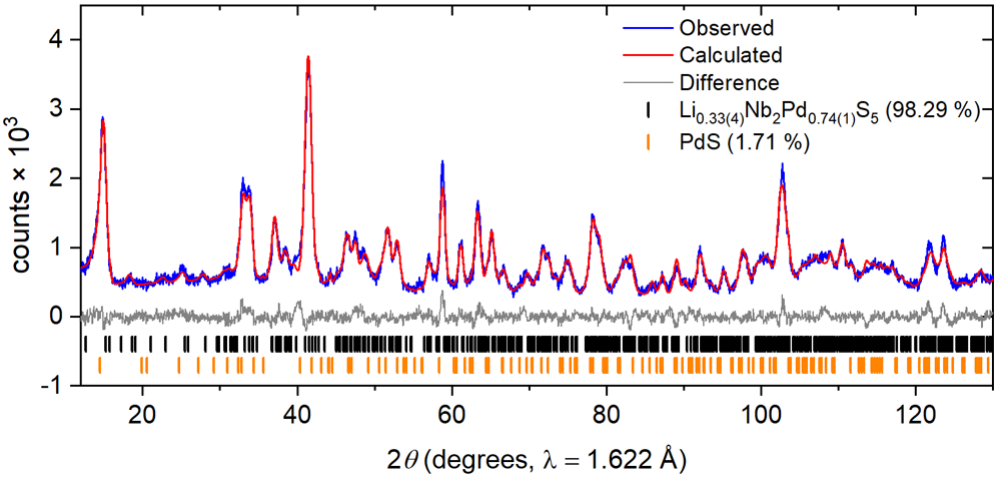
Rietveld refinement of the neutron diffraction pattern on Li_0.33(4)_Nb_2_Pd_0.74(1)_S_5_. *R*_wp_: 3.26%

**Figure 8:**
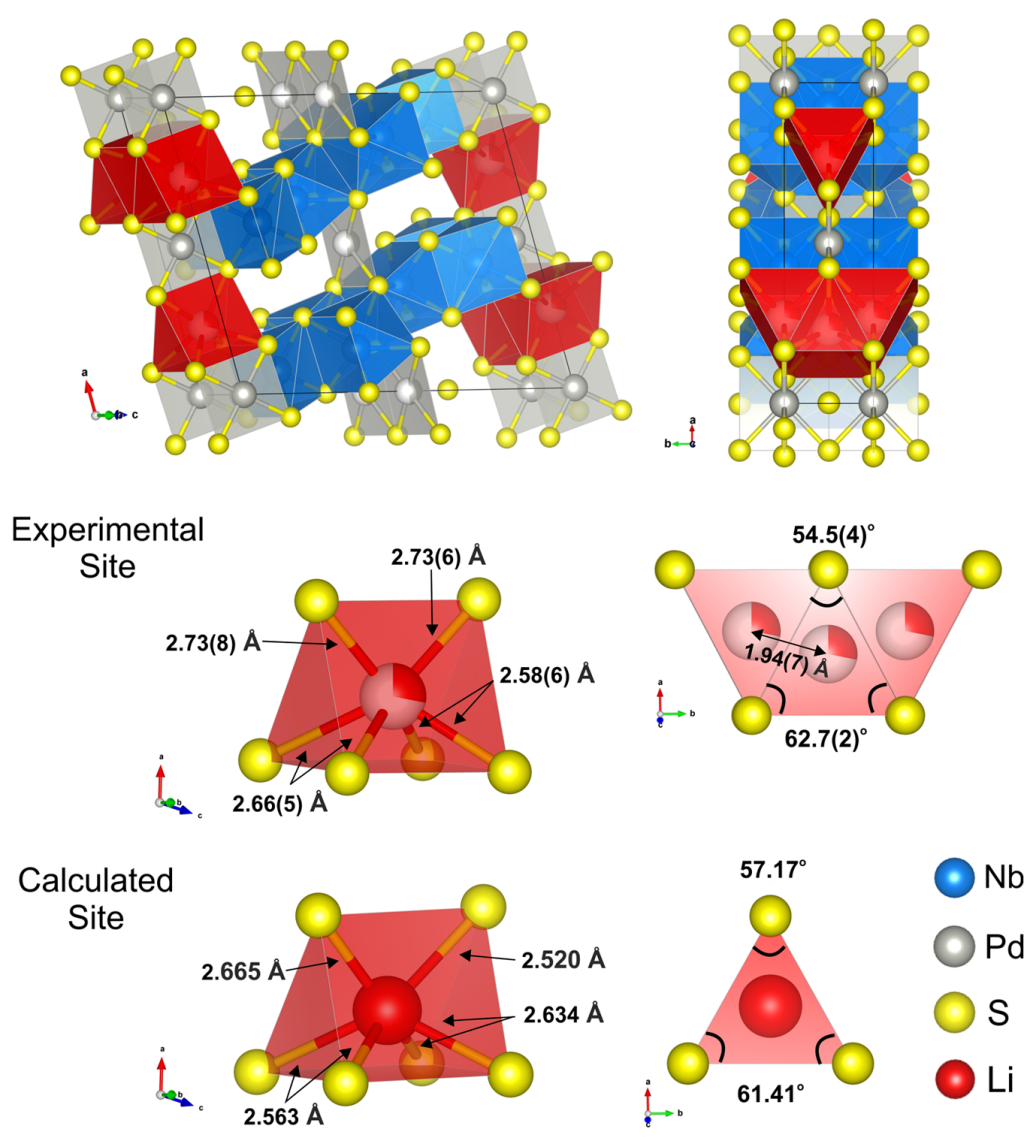
Schematic of the trigonal prismatic Li site obtained experimentally from the refinement of neutron diffraction data and obtained theoretically from DFT calculations.

Room-temperature powder neutron diffraction measured on Echidna, ANSTO, was used on the same sample as for the PXRD analysis to determine Li positions since the scattering of X-rays by Li is so weak compared to that of the much heavier 4d metals. Li is a negative scatterer with a coherent scattering length of −1.9 fm,^36^ and a negative scattering center was located using a difference Fourier map at (0.208(5), 0.5, 0.002(3)) which corresponds to a triangular prismatic site with the base of the prism consisting of one edge each of two adjacent Pd(1)S_4_ units stacked in the *b* direction (Figure 8). This site was refined to have an occupancy of 0.33(4) Li per formula unit. The Pd(2) occupancy was refined to 0.47(2), which was similar to the consistent occupancies of 0.496(3) and 0.485(3) obtained from the PXRD refinement of the parent and intercalate, respectively. Allowing for the uncertainty in the refined parameters and the approximate 1:3 ratio of the magnitudes of the scattering lengths of Li and Pd, this suggests that the Li is not intercalated into the vacant Pd(2) linkage sites above the 5% level.

Structural information is provided in Table 2. Thermal displacement parameters were constrained to be isotropic and equal for similar elements and sites to avoid excessive parameter correlation, particularly in refinements against the lower-resolution neutron powder diffraction data. The Li position and occupancy have higher degrees of uncertainty. This is most likely due to its weak scattering length (−1.9 fm) relative to that of the other elements (Nb = 7.05 fm; Pd = 5.91 fm; and S = 2.85 fm), relatively low occupancy, and the possibility that Li is mobile in the space between the layers.

**Table 2:** Positional Parameters of Li_0.33(4)_Nb_2_Pd_0.74(1)_S_5_ Obtained from the Refinement of Synchrotron X-ray Diffraction and Neutron Diffraction^a^

**Li**_**0.33(4)**_**Nb**_**2**_**Pd**_**0.74(1)**_**S**_**5**_**(*****Z*****= 4,RMM= 428.2(3) g mol**^**–1**^**)**	
**Diffractometer**	I11 (PXRD) [Echidna (PND)]
**Wavelength/Å**	0.824970(5) [1.622]
***d-*****Space** **Range/Å**	1.1–18.9 [2.1–9.3]
**Temperature/K**	300
***R*_wp_**	2.79 [3.26]
***R*_p_**	1.58 [2.54]
***χ*^*2*^**	6.56 [1.39]
**Crystal System**	Monoclinic
**Space Group**	*C*2/*m* (No. 12)

***a*/Å**	***b*/Å**	***c*/Å**	**Volume/Å^3^**	**β/deg**
12.3390(5)	3.2933(1)	15.3447(6)	598.03(4)	106.444(1)
[12.3428(12)]	[3.2914(2)]	[15.3390(15)]	[597.5(1)]	[106.489(7)]

**Positional** **Parameters**						
**Atom**	***x***	***y***	***z***	**Occupancy**	**Wyckoff Symbol**	***U*_iso_/Å^2^**
Nb(1)	0.0737(2) [0.0779(6)]	0.5	0.1852(2) [0.1868(4)]	1	4*i*	0.0022(8) [0.0042(8)]
Nb(2)	0.1475(2) [0.1443(5)]	0	0.3768(2) [0.3777(4)]	1	4*i*	0.0019(7) [0.0042(8)]
Pd(1)	0	0	0	1	2*a*	0.0031(8) [0.0042(8)]
Pd(2)	0	0	0.5	0.485(3) [0.470(17)]	2*c*	0.0031(8) [0.0042(8)]
S(1)	0.3579(6) [0.3594(10)]	0	0.4949(5) [0.5031(9)]	1	4*i*	0.0011(7) [0.0086(16)]
S(2)	0.2517(6) [0.2506(14)]	0.5	0.3116(5) [0.3054(11)]	1	4*i*	0.0011(7) [0.0086(16)]
S(3)	0.1701(6) [0.1612(11)]	0	0.1140(5) [0.1155(9)]	1	4*i*	0.0011(7) [0.0086(16)]
S(4)	0.4165(6) [0.4214(13)]	0.5	0.1256(5) [0.1201(10)]	1	4*i*	0.0011(7) [0.0086(16)]
S(5)	0.4953(6) [0.4981(12)]	0	0.3075(5) [0.3155(9)]	1	4*i*	0.0011(7) [0.0086(16)]
Li	[0.208(5)]	0.5	[0.002(4)]	[0.33(4)]	4*i*	[0.0042(8)]

a Values from neutron refinement are given in [].

### 3.3 Computational Analysis

**Figure 9:**
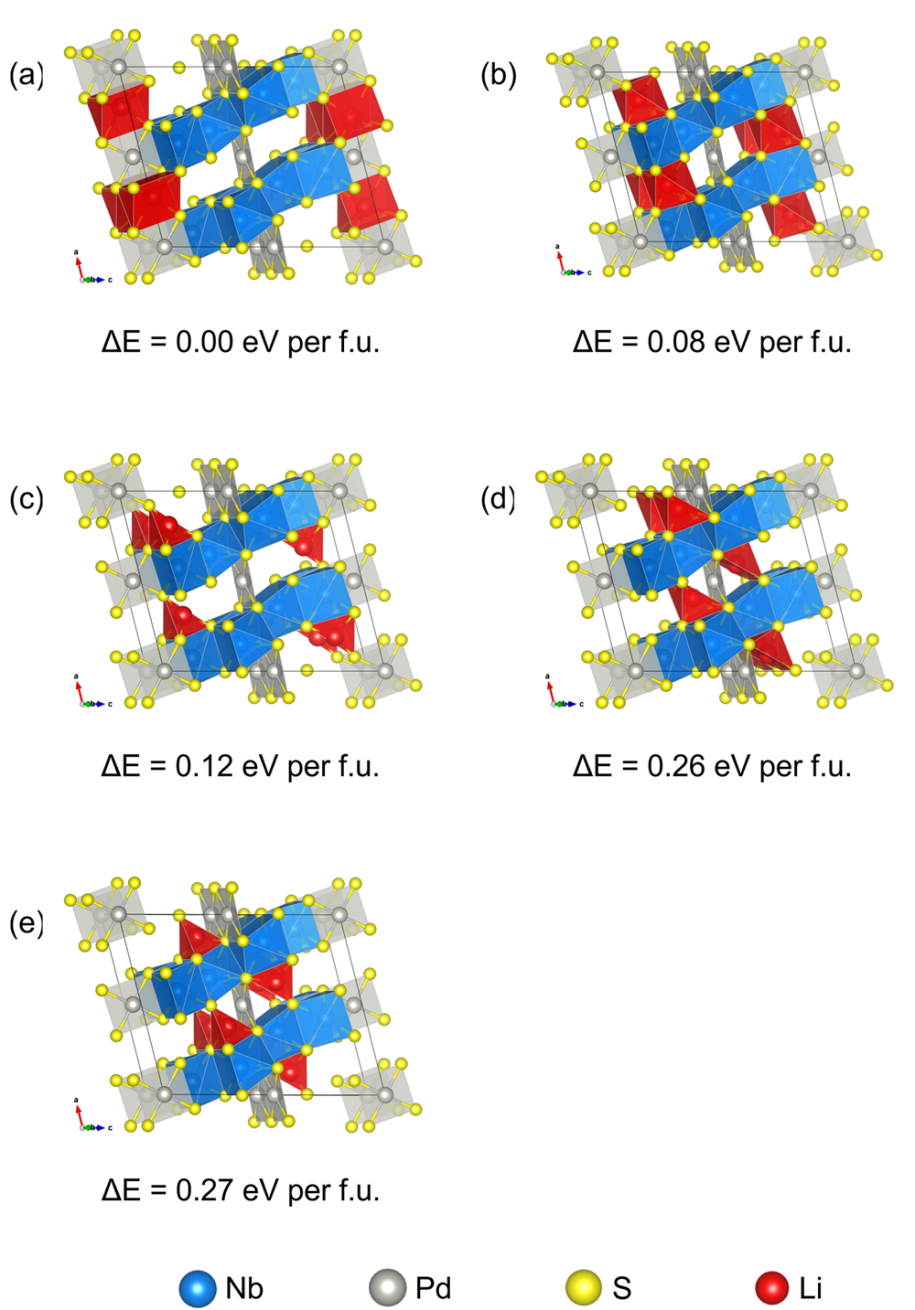
Schematic of the Li sites (a–e) determined from DFT ranked from lowest energy to highest.

DFT calculations using the stochiometric *C*2/*m* Nb_2_PdS_5_ model discussed above were used to locate all possible Li sites within the compound and to calculate their relative energies. One Li atom was placed in the 32-atom supercell with four formula units of Nb_2_PdS_5_, corresponding to a composition of Li_0.25_Nb_2_PdS_5_, similar to that deduced experimentally. Twenty-two unique interstitial sites were identified, and each Li interstitial structure was allowed to relax. All Li interstitials were relaxed to five distinct possible sites. The calculated lattice parameters of the stoichiometric Nb_2_PdS_5_ (parent) are similar to that of the experimental parent Nb_2_Pd_0.74(1)_S_5_, with a maximum expansion (1.5%) along the *c* axis and a small increase in the monoclinic angle. The volume of the calculated Nb_2_PdS_5_ cell is only ∼0.3% larger than the experimental parent cell. Upon incorporating Li, the cell volume was predicted to increase by 0.54%, which is smaller than the volume expansion observed between the experimental structures (2.22%). A diagram showing the locations of the five Li sites calculated after relaxation is shown below in Figure 9, with the computed structural information given in Table 4.

**Table 3:** Lattice Parameters of Nb_2_Pd_0.74_S_5_, the Experimentally Determined Intercalated Phase, and DFT Geometry-Optimized Structures

**Compound**	**Nb**_**2**_**Pd**_**0.74(1)**_**S**_**5**_	**Li**_**0.33(4)**_**Nb**_**2**_**Pd**_**0.74(1)**_**S**_**5**_	**Nb**_**2**_**PdS**_**5**_	**Li**_**0.25**_**Nb**_**2**_**PdS**_**5**_
	Experimental Parent	Experimental Intercalate	Calculated Parent	Calculated Intercalate
***a*****/Å**	12.1448(1)	12.3390(5)	12.1434	12.1897
***b*****/Å**	3.27971(2)	3.2933(1)	3.2723	3.2825
***c*****/Å**	15.0798(1)	15.3447(6)	15.3027	15.3662
**Volume/Å**^**3**^	585.04(1)	598.03(4)	587.0197	590.1944
**β/deg**	103.161(9)	106.444(1)	105.1257	106.2761

**Table 4:** Description of Li Sites (a)–(e) Depicted in Figure 9

**Site**		**(a)**	**(b)**	**(c)**	**(d)**	**(e)**
**Δ*****E*****/eV**		0.00	0.32	0.50	1.03	1.09
**Site**		4*i*	4*i*	4*i*	4*i*	4*i*
**Li SiteEnergy/eV f.u.**^**–1**^		0.00	0.08	0.12	0.26	0.27
**Site Coordinates**	***x***	0.2208	0.1621	0.1852	0.0937	0.1266
	***y***	0.5	0.5	0	0	0.5
	***z***	0.9941	0.7991	0.8087	0.6179	0.6366
**Mean**Li–S Bond Length/Å		2.596	2.457	2.326	2.435	2.242
**Description of the Coordination Environment**		Triangular prism	Octahedral	Seesaw-like/Distorted Tetrahedral	Square pyramidal	Seesaw-like/Distorted Tetrahedral

The lowest energy site (site (a)) calculated was the triangular prismatic site which was identified independently as the occupied site from the neutron refinement. This site has a calculated mean Li–S bond length of ∼2.6 Å. Typical Li–S distances seen for 6-coordinate Li sites in intercalates such as Li_0.63_NbS_2_ and LiIrS_2_ have been reported to be 2.55 and 2.67 Å, respectively.^37,38^ Therefore, the calculated bond distances are in line with previous reports. Note that the calculated energies using a composition similar to that deduced experimentally avoid close Li–Li contacts. If the site in Figure 9(a) were to be fully occupied, then adjacent Li ions would be in very close proximity to each other (∼1.8 Å), which would be electrostatically unfavorable. Therefore, the low occupancy found experimentally is consistent with this.

The second lowest energy site (site (b)) calculated is an octahedral (i.e., triangular antiprismatic) site using the Nb(1)S_6_ prism faces with an average Li–S bond length of 2.46 Å, a distance more in line with typical Li–S distances seen for Li_2_S consisting of tetrahedral Li environments with bond lengths of 2.48 Å.^39^ Site (c) can be viewed as a seesaw-like or distorted tetrahedral environment with smaller-than-expected Li–S distances of ∼2.33 Å. The shorter bond lengths and the strained geometry increase the energy of this site. Although site (d) is a square-based pyramid site with a reasonable mean Li–S bond length, the distance of Li to the Pd(2) site is only 1.9 Å. The proximity of the two positive charge centers causes repulsion, hence the energy is much higher. Finally, site (e) is also a highly distorted tetrahedral or seesaw-like coordination with smaller bond lengths of 2.24 Å and proximity to Pd(2) of ∼2.8 Å; the combination of these factors makes this site the highest in energy.

### 3.4 Structural Changes Following Li Intercalation

**Figure 10:**
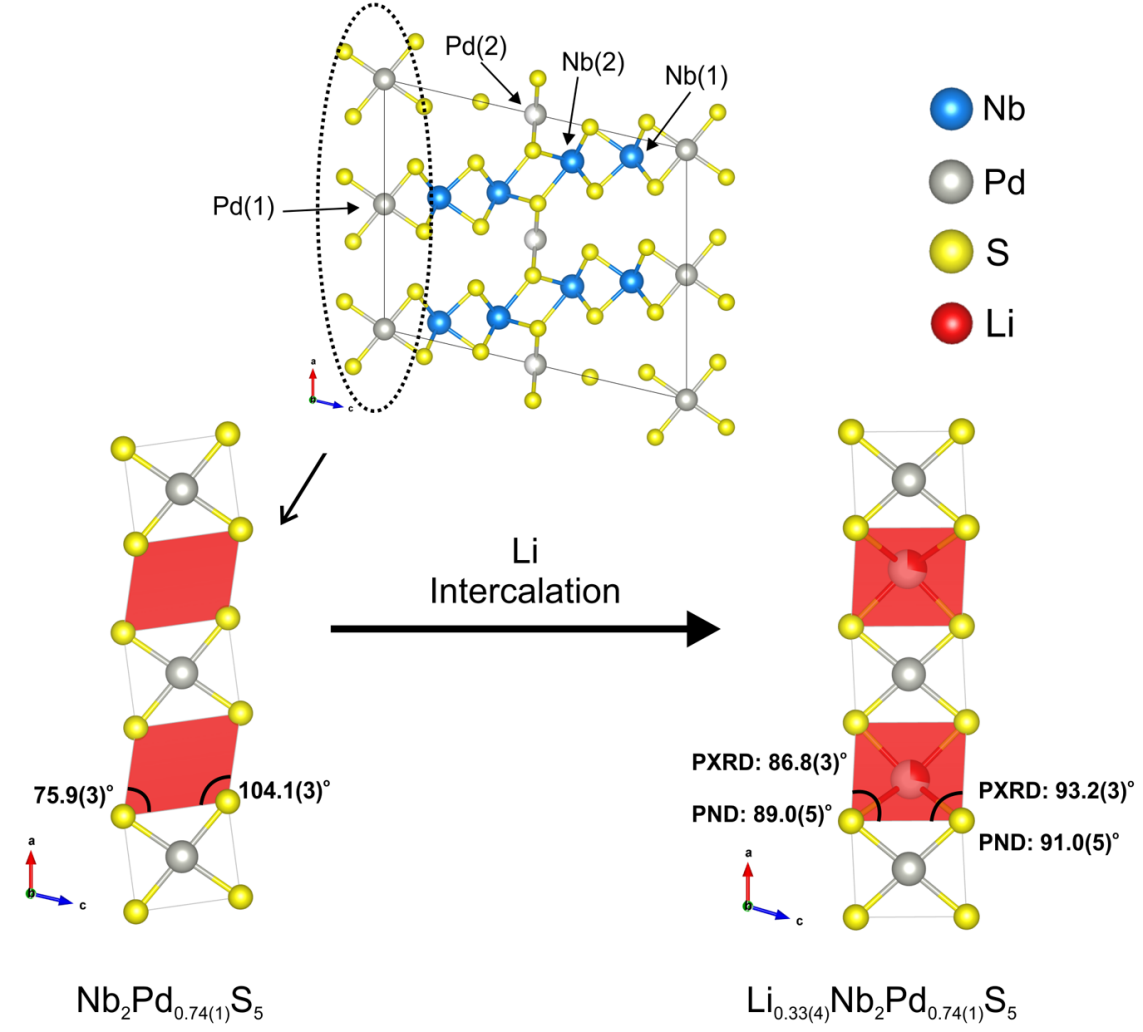
Schematic diagram of the Li site before and after intercalation showing the rotation of the Pd(1)S_4_ units leading to the triangular prismatic Li site becoming less distorted after the Li is intercalated.

Occupancy of the triangular prismatic Li site shown in Figure 8 and Figure 9(a) is evident from the neutron refinement and is corroborated by the computation. Before intercalation, the empty triangular prismatic site is distorted, with the prism being distorted such that the angles between the rectangular face which is approximately perpendicular to the *a* axis and the triangular faces deviate by ±14.1(3)° from the undistorted value of 90°, as shown in Figure 10. (See also Figure 8 for a perspective diagram of the site.) After intercalation, the layers undergo a relative motion which involves a rotation of the Pd(1)S_4_ square planar units about a direction parallel to the *b* axis so that the triangular prisms become less distorted with the interfacial angle deviating by only ±3.2(3) and ±1.0(5)° from the ideal 90° in the X-ray and neutron models, respectively.

A closer examination of the Li site is shown in Figure 8; the Li is distorted within the prism, with Li–S bond lengths ranging from 2.58(6) to 2.73(8) Å, giving a mean bond length of 2.66(3) Å. Although this is slightly longer than typical Li–S distances reported (2.55 Å for Li_0.63_NbS_2_),^37^ it is in line with that reported for LiIrS_2_.^38^ The bond valence sum calculated for Li was 0.88(8), in line with the oxidation state of Li^+^ being + 1 (calculations were performed using tabulated values from Brown and Altermatt).^40^ The triangular prisms are stacked, sharing rectangular faces in the *b* direction and resulting in adjacent centers of these prisms being in close proximity to one another, requiring a Li–Li distance of 1.94(7) Å were the site to be fully occupied. Therefore, while the occupation of this site is favored over the occupation of other sites in the calculations, full occupancy may be difficult to obtain experimentally due to the Li–Li repulsions, which is consistent with the substoichiometric amount of Li needed in the synthesis to avoid the formation of impurity phases; the 0.33(4) occupancy obtained from the neutron refinement is thus reasonable since it minimizes Li–Li repulsion by avoiding the need to populate adjacent sites. The sharing of rectangular faces by the triangular prisms may lead to high Li ion mobility.

### 3.5 Li Deintercalation

**Figure 11:**
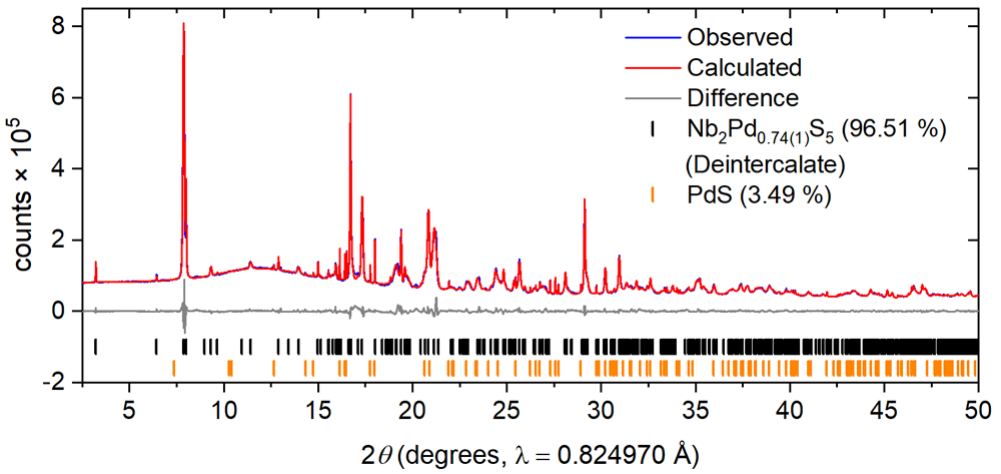
Synchrotron X-ray refinement of deintercalated Li_0.33(4)_Nb_2_Pd_0.74(1)_S_5_ back to Nb_2_Pd_0.74(1)_S_5_. *R*_wp_: 3.14%.

### 3.6 Magnetometry

**Figure 12:**
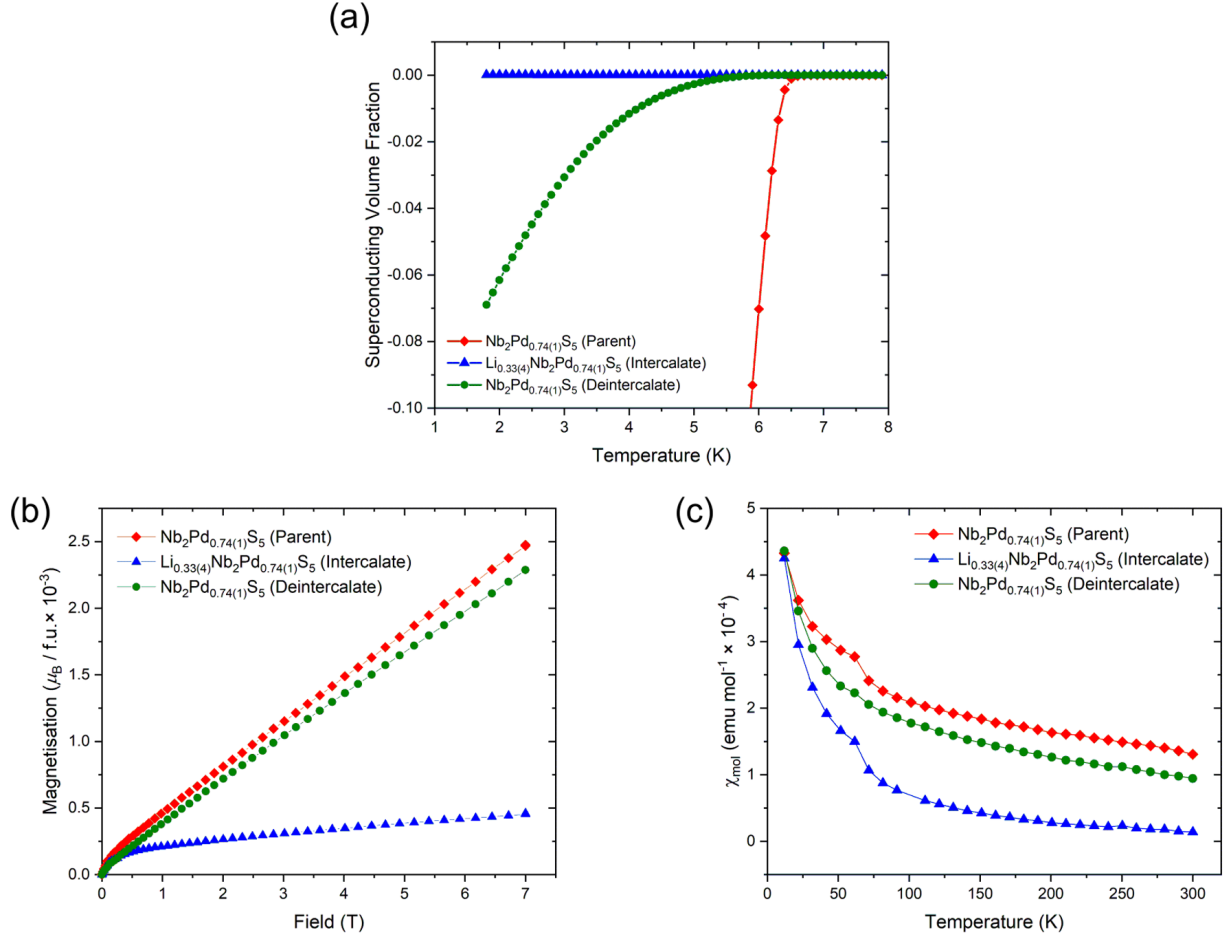
(a) Superconducting volume fraction against temperature for zero-field-cooled measurements for a field of 10 Oe. (See also Figure 3 for the behavior of the Nb_2_Pd_0.74(1)_S_5_ host material.) (b) Magnetization against field and (c) molar susceptibility (χ_mol_) against temperature of parent Nb_2_Pd_0.74(1)_S_5_ and the intercalated and deintercalated phases. Molar susceptibility was measured at 3 and 4 T with the difference taken between them to eliminate ferromagnetic impurities that are evident from the curvature at a low field in (b).

Li intercalation causes suppression of the superconductivity with no observable superconducting transition evident down to 2 K. Superconductivity is observed again upon deintercalation back to the original parent phase. However, it is only partially restored, consistent with the observation that the original lattice parameters are not fully recovered. The onset transition temperature in the material obtained by Li deintercalation is lower (*T*_c_ ≈ 5.5 K) than in the original host phase (Figure 12(a)), and the superconducting volume fraction is much reduced (∼7% compared to 80% in the original host material prior to lithiation). This could be potentially due to a trace amount of Li still present in the deintercalated sample.

The parent Nb_2_Pd_0.74(1)_S_5_ compound is paramagnetic with a room-temperature molar susceptibility (*χ*_mo__l_) of 1.865(3) × 10^–4^ emu mol^–1^. Variable-temperature measurements showed that the susceptibility does not obey the Curie–Weiss law but could be fit by a temperature-independent contribution (arising from Pauli paramagnetism and core diamagnetism) and a small Curie-type contribution. The susceptibility plot can be fitted to the equation , where *χ*_0_ is the temperature-independent susceptibility and  is the Curie contribution arising from small amounts of impurity or localized spin states (∼0.05 *S* = 1/2 impurity spins per mole) (the fits are shown in Figure S2). *χ*_0_ consists of a Pauli paramagnetic contribution and a second temperature-independent component, *χ*_D_, arising from the diamagnetism of the core electrons. For Nb_2_Pd_0.74(1)_S_5_, *χ*_D_ is calculated to be approximately −1.865(3) × 10^–4^ emu mol^–1^ by summing the individual diamagnetic components of each ion with the values obtained from Bain et al.^41^ Subtracting *χ*_D_ from *χ*_0_ gives the estimated Pauli paramagnetic susceptibility *χ*_P_. The *χ*_P_ values are given in Table 5.

**Table 5:** Susceptibility Values of Nb_2_Pd_0.74(1)_S_5_ Host Material and the Intercalated and Deintercalated Phases

	**Nb**_**2**_**Pd**_**0.74(1)**_**S**_**5**_	**Li**_**0.33(4)**_**Nb**_**2**_**Pd**_**0.74(1)**_**S**_**5**_	**Nb**_**2**_**Pd**_**0.74(1)**_**S**_**5**_
	Parent	Intercalate	Deintercalate
***χ***_**0**_**/emu mol**^**–1**^	8.8(4) × 10^–5^	–2.7(3) × 10^–5^	6.3(4) × 10^–5^
***χ***_**D**_**/emu mol**^**–1**^	–1.865(3) × 10^–4^	–1.868(3) × 10^–4^	–1.865(3) × 10^–4^
***χ***_**P**_**/emu mol**^**–1**^	2.75(4) × 10^–4^	1.60(3) × 10^–4^	2.50(4) × 10^–4^

**Figure 13:**
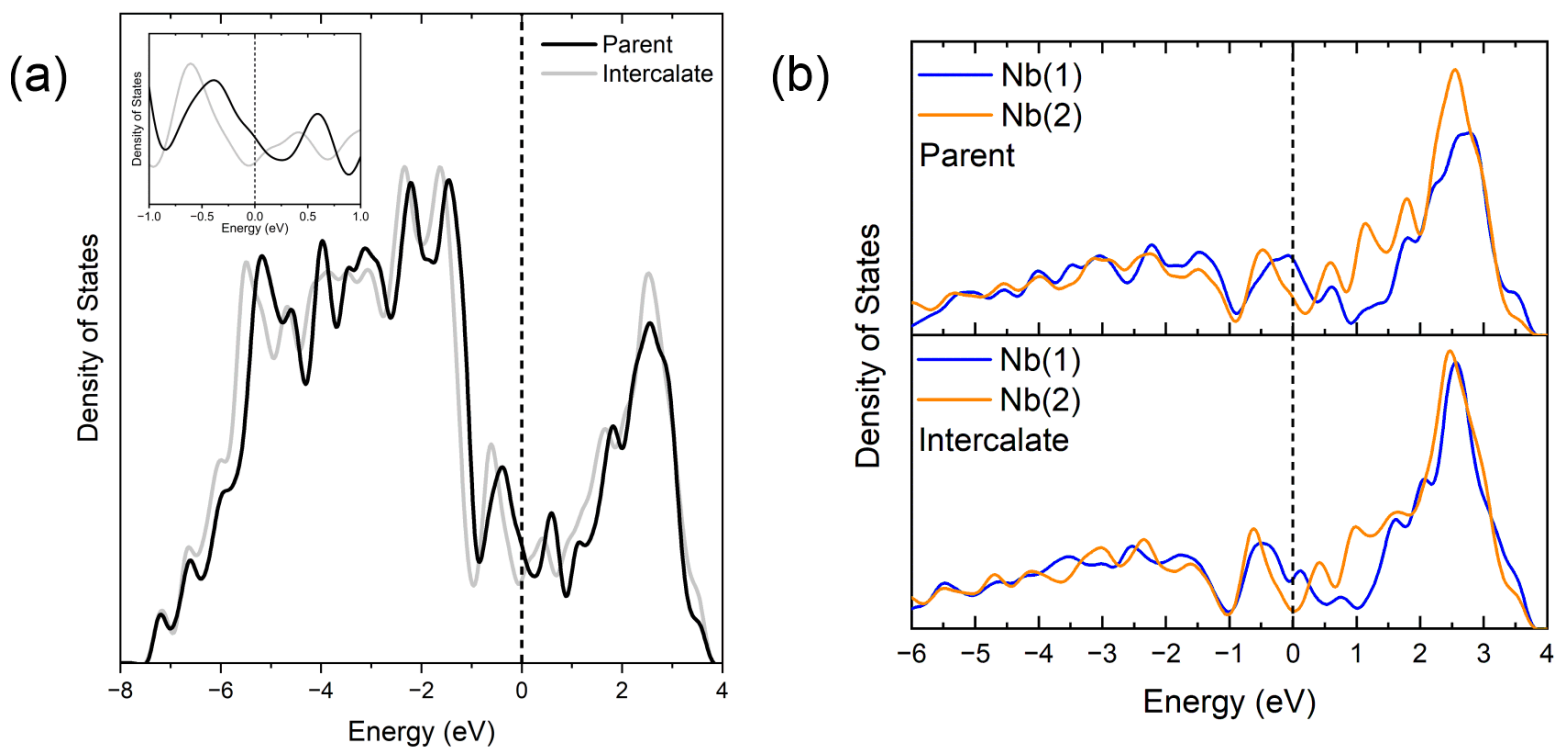
(a) Comparison of the total density of states of the parent Nb_2_PdS_5_ and intercalate Li_0.25_Nb_2_PdS_5_ calculated using the lowest-energy Li site. The inset shows a close-up around the Fermi level with a lower density of states for the intercalate compared to the parent at *E*_F_. (b) Projected density of states for Nb 4d orbitals for the parent Nb_2_PdS_5_ and intercalate Li_0.25_Nb_2_PdS_5_ calculated using the lowest-energy Li site.

Upon intercalation, *χ*_mol_ decreases to 2.03(2) × 10^–5^ emu mol^–1^, a reduction by an order of magnitude. *χ*_P_ decreases from 2.75(4) × 10^–4^ to 1.60(3) × 10^–4^ emu mol^–1^, a reduction of approximately 40%. This therefore shows that the density of states at the Fermi level, which is proportional to the Pauli susceptibility, is reduced. Figure 13(a) shows a comparison of the density of states between Nb_2_PdS_5_ and Li_0.25_Nb_2_PdS_5_ (calculated using the lowest-energy Li site) which depicts a clear shifting of the Fermi level to a lower density of states upon intercalation. (See Figure S3 for full density of states and band structure of Li_0.25_Nb_2_PdS_5_.) A greater change in the partial density of states at *E*_F_ is observed on the Nb 4d bands (Figure S3), indicating that Nb^4+^ is partially reduced upon intercalation. Figure 13(b) shows the reduction to be more localized on the Nb(1) site. This is consistent with the fact that the most probable Li site is closer to Nb(1) than Nb(2). Li intercalation changes the structure and bonding in the vicinity of the triangular prismatic Li site. The differences in the density of states and band structure before and after Li intercalation are due to a combination of structural changes as well as electrons being added. This may also explain the suppression of the superconductivity since *T*_c_ increases with increasing density of states at *E*_F_ in the BCS theory. Shen et al. reported on the substitution of Pd in Nb_2_Pd_0.76_S_5_ by Ag or Ir and concluded that Ir substitution (hole doping) increases *T*_c_ with a maximum at 40% substitution and then superconductivity is suppressed with increasing Ir content.^16^ Conversely, Ag substitution (electron doping) decreases *T*_c_ with *T*_c_ < 2 K at 20% Ag substitution and complete suppression at 40% substitution. This would appear consistent with navigating the peak in the DOS found in the calculations (Figure 4(a)) and in previous reports.^35^ A phase diagram showed a superconducting dome in which Shen et al. drew similarities to that seen for the cuprate and iron pnictide superconductors.^42,43^ Li intercalation mimics Ag substitution, in which the system is injected with electrons. A Li content of 0.33(4) would therefore be analogous to 30–40% Ag doping, so the full suppression of superconductivity on Li intercalation is quantitatively consistent with other reported changes in the electron count.

Deintercalation is shown to increase the *χ*_mol_ and *χ*_P_ back to values similar to those of the initial host material with values of 1.775(9) × 10^–4^ and 2.50(4) × 10^–4^ emu mol^–1^, respectively. *χ*_P_ is ∼10% smaller for the deintercalated phase than for the parent phase, which corresponds to a slightly lower density of states at the Fermi level than in the parent, and this is consistent with the partial restoration of the superconductivity at lower *T*_c_ and volume fraction, as seen in Figure 12. Assuming *χ*_P_ follows a linear trend with composition, a *χ*_P_ of 2.50(4) × 10^–4^ emu mol^–1^ would correspond to a Li content of ∼0.07, consistent with the unit cell volume changes. A Li content of ∼0.07 can be approximated to 10% Ag doping reported by Shen et al., in which they report a *T*_c_ of ∼4 K. Given the relatively low scattering length of Li compared with that of the other elements, this level of remnant Li is difficult to locate quantitatively, and as noted above, we cannot rule out, given intrinsic experimental uncertainties, a scenario where a few percent of Li becomes intercalated into vacancies at the Pd(2) site and is then not removed on deintercalation.

### 3.7 Electrochemical Li Intercalation

**Figure 14:**
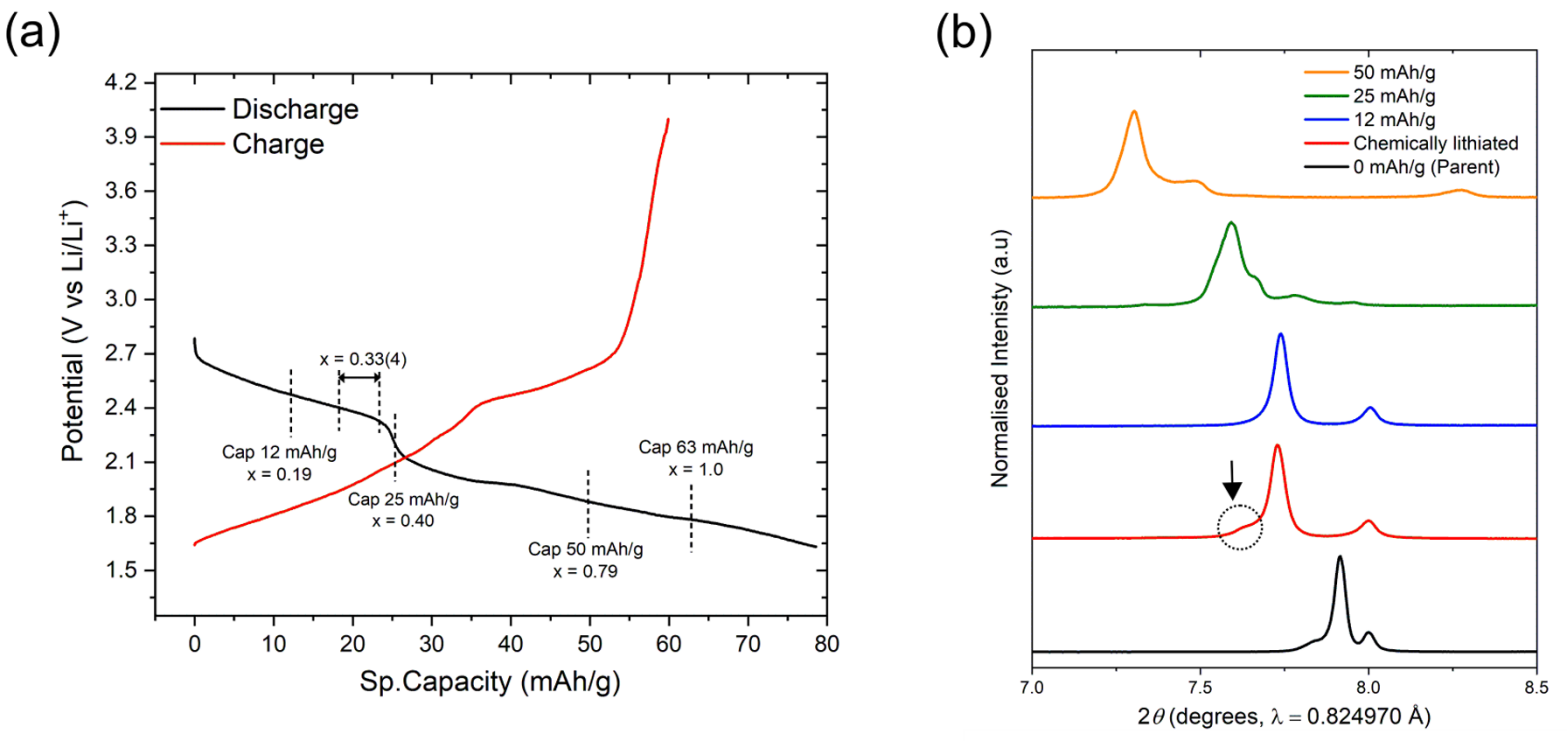
(a) Plot of potential against specific capacity with specific capacities of 12, 25, 50, and 63 mAh/g indicated, which correspond to theoretical Li contents of 0.19, 0.40, 0.79, and 1, respectively. The Li content of 0.33(4) obtained from the refinement of the chemically lithiated phase is shown as a reference. (b) X-ray diffraction pattern of the main 201 and 200 Bragg reflections with varying starting Li stoichiometries. The arrow shows that the chemically synthesized phase contains a small amount of a second intercalate phase (not present in the 12 mAh/g sample) that dominates at higher Li contents.

Li was intercalated electrochemically into Nb_2_Pd_0.74(1)_S_5_ for comparison with the chemically lithiated material, allowing more control of the extent of the intercalation and the attempt to achieve a higher Li content without the formation of side phases. The potential vs specific capacity graph in Figure 14(a) shows a step in the discharge curve at around 25–30 mAh/g, indicating new phase formation. The parent compound was discharged to three distinct points: 12, 25, and 50 mAh/g corresponding to theoretical Li contents (*x* in Li_*x*_Nb_2_Pd_0.74_S_5_) of 0.19, 0.4, and 0.79, respectively. Here a theoretical Li content of *x* = 1 would be achieved when discharging to 63 mAh/g. The Li content of 0.33(4) obtained chemically from the neutron refinement is analogous to a specific capacity of 18.3–23.3 mAh/g (as indicated in Figure 14(a)), corresponding to the region just prior to the step in the discharge curve. The PXRD patterns of three electrochemically intercalated samples were measured on the I11 beamline. Figure 14(b) shows that the 201 Bragg reflection moves to a higher *d* spacing with a greater degree of intercalation as expected.

**Table 6:** Lattice Parameters of the Main Phases in the Electrochemical Intercalation

**Compound**	**Nb**_**2**_**Pd**_**0.74(1)**_**S**_**5**_	**Li**_**0.33(4)**_**Nb**_**2**_**Pd**_**0.74(1)**_**S**_**5**_	**Li**_*x*_**Nb**_**2**_**Pd**_**0.75(1)**_**S**_**5**_	**Li**_*x*_**Nb**_**2**_**Pd**_**0.75(2)**_**S**_**5**_	**Li**_**x**_**Nb**_**2**_**Pd**_**x**_**S**_**5**_^a^
	Parent	Chemical (Li/NH_3_)	Electrochemical 12 mAh/g	Electrochemical 25 mAh/g	Electrochemical 50 mAh/g
***a*****/Å**	12.1448(1)	12.3390(5)	12.3266(3)	12.6208(4)	13.0426(10)
***b*****/Å**	3.27971(2)	3.2933(1)	3.29196(6)	3.30577(8)	3.3295(1)
***c*****/Å**	15.0798(1)	15.3447(6)	15.3412(7)	15.2049(4)	15.5651(8)
**Volume/Å**^**3**^	585.04(1)	598.03(4)	597.32(3)	611.61(3)	640.05(7)
**β/deg**	103.161(9)	106.444(1)	106.360(9)	105.396(1)	108.751(4)
**Mean Bond**Lengths/Å					
**Nb(1)–S**	2.469(3)	2.463(3)	2.463(3)	2.461(3)	–
**Nb(2)–S**	2.523(3)	2.545(3)	2.530(3)	2.556(4)	–
**Pd(1)–S**	2.358(5)	2.373(6)	2.377(4)	2.363(6)	–
**Pd(2)–S**	2.403(5)	2.390(6)	2.423(3)	2.470(6)	–

a Note that for this sample, only a model-independent fit to give the lattice parameters of the two phases was possible, so no structural details were extracted.

**Figure 15:**
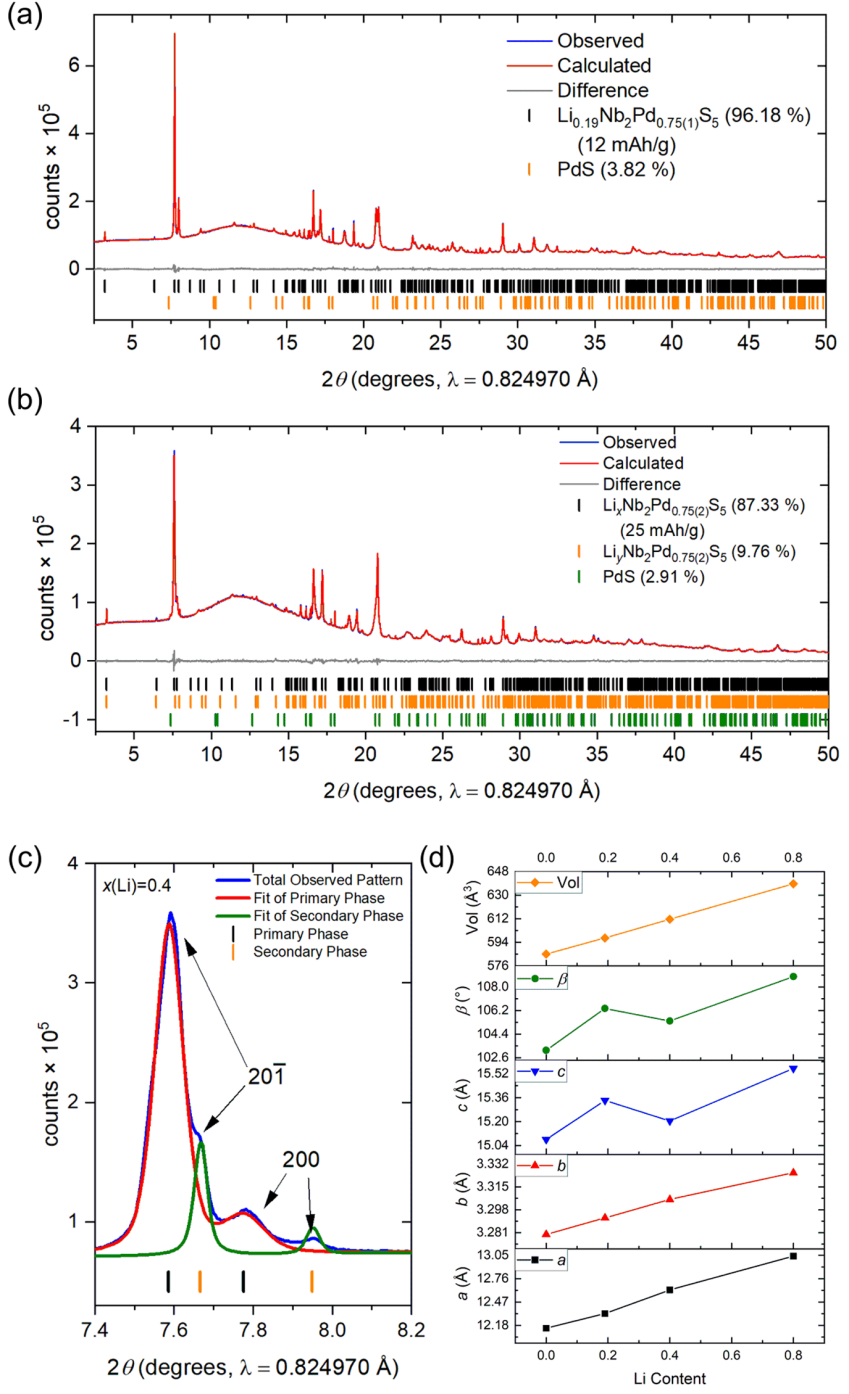
(a, b) Rietveld refinement of the electrochemical intercalates for the samples discharged to 12 and 25 mAh/g with *R*_wp_ values of 1.23 and 1.00%, respectively. (c) Main 201 and 200 Bragg reflections from the refinement in (b) for the sample discharged to 25 mAh/g showing the phase separation. (d) Plot of lattice parameters against Li content showing a linear increase in the unit cell volume with Li content derived from the specific capacity.

The sample discharged to 12 mAh/g produced a single-crystalline phase with structural parameters similar to those of the chemically intercalated phase with a difference in the volume of only ∼0.1%. Examining the diffraction pattern of the intercalated sample discharged to 25 mAh/g (*x*(Li) = 0.4), at the step in the discharge curve, the compound has expanded significantly but has also undergone phase separation, and the pattern can be fit as two intercalated phases, as shown in Figure 15(c). There is evidence from the diffraction pattern of the chemical intercalate Li_0.33(4)_Nb_2_Pd_0.74(1)_S_5_ that the second phase starts to form, and this was clear when larger amounts of Li were used in the chemical intercalation (Figure 5). The main phase (∼87% by mass) in the sample discharged to 25 mAh/g has expanded by approximately 0.48, 0.03, and 0.13 Å on the *a*, *b*, and *c* axes, respectively, compared to the parent with a 2.2° increase in the monoclinic angle. Compared to the intercalate discharged at 12 mAh/g and the chemical intercalate, the unit cell volume of the sample discharged to 25 mAh/g has increased by 2.4%. The main expansion compared to the sample discharged to 12 mAh/g is in the *a* axis with an ∼0.27 Å increase which is reflected in the increase in the Pd(2)–S bond length. The *c* axis has seen a contraction of ∼0.14 Å, with the monoclinic angle also reducing by ∼1°. The minority intercalated phase in the sample discharged to 25 mAh/g has lattice parameters of *a* = 12.4318(5) Å, *b* = 3.3003(2) Å, *c* = 15.3627(9) Å, and β = 106.780(4)°; therefore, this phase is more akin to the sample discharged to 12 mAh/g but with an ∼1% volume increase and to the chemically synthesized sample. As stated earlier, due to the proximity of the centers of the Li-containing triangular prism sites to one another, they cannot be occupied with high Li content. It is likely that the 0.33(4) Li content obtained from the neutron refinement for the chemically synthesized sample is the upper limit to the occupancy of the triangular prism site before it becomes energetically unfavorable. This is consistent with *x*(Li) = 0.33(4) corresponding to the region just before the step in the discharge curve.

This significant change in the structure evident for the sample discharged to 25 mAh/g, i.e., *x*(Li) = 0.40, suggests the possibility of extra Li occupying another site. When examining the changes in the triangular prismatic site upon intercalation, it can be seen that at low Li content (*x*(Li) = 0.19) the site becomes less distorted in a manner similar to that of the chemically intercalated compound. However, at higher Li content (*x*(Li) = 0.4), the site becomes distorted again, indicating that the layers shift, perhaps in order to accommodate Li in another site.

**Figure 16:**
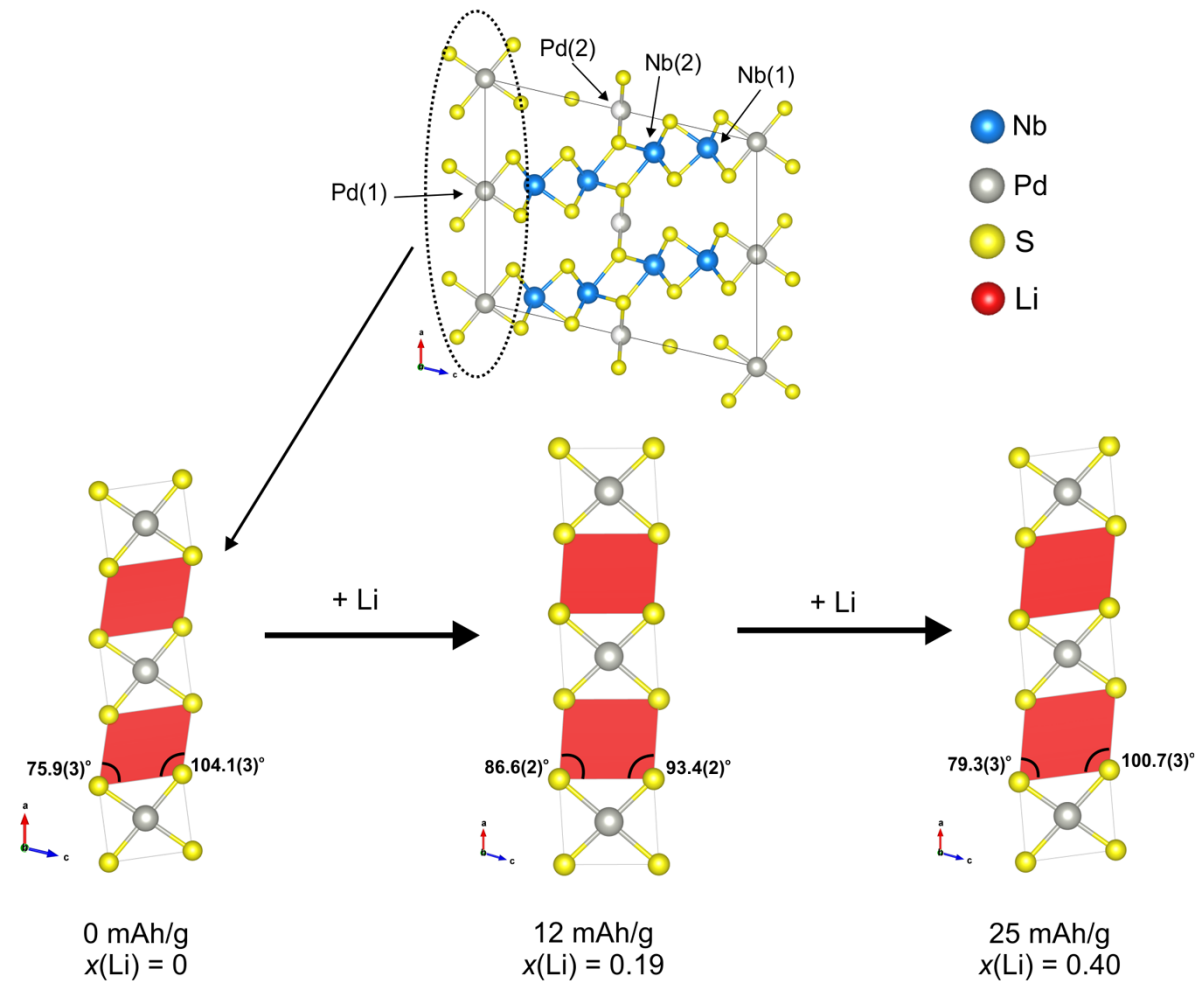
Schematic of the Li triangular prism site before and after intercalation showing how at low Li content the site becomes less distorted, but at higher Li content, the site distorts again, suggesting that the layers are shifting to accommodate Li in another site.

From the DFT calculations, the triangular prismatic site was identified as the lowest energy site for the model Li_0.25_Nb_2_PdS_5_, and the diffraction measurements bear this out. However, the Li site energies will change upon inserting more Li into the structure, as Li ions can no longer be considered to be independent of one another. The structural change shown in Figure 16 for the 25 mAh/g sample may result from the layers accommodating Li in the new site, such as the octahedral site with intersite distances of ∼3.3 Å (equal to the *b* axis) and thus higher potential Li occupancy. Neutron diffraction on the electrochemically synthesized phases would be required to explore this further, but this is challenging on account of the volume of sample needed and the inevitable background arising from the additives (binder, etc.) mixed with the phase for the electrochemical synthesis. Our attempts to use excess Li/NH_3_ or other reagents in the chemical synthesis produced multiphase products, so electrochemical synthesis seems more promising for the Li-rich phases, although multiphase samples are also evident in that case as described.

Phase separation remains when the cell is discharged to 50 mAh/g (*x* = 0.79 in Li_*x*_Nb_2_Pd_0.74_S_5_). The main phase was indexed to lattice parameters *a* = 13.042(1) Å, *b* = 3.3295(1) Å, *c* = 15.5651(8) Å, and β = 108.751(4)°; an even greater increase in the unit cell compared to that of the sample discharged to 25 mAh/g (a volume increase of ∼4.6% and a volume increase of ∼9.4% over the original Nb_2_Pd_0.74(1)_S_5_ host phase). The increase in the volume follows a linear trend with increasing Li content, as shown in Figure 15(d). The secondary phase was indexed to lattice parameters *a* = 12.858(1) Å, *b* = 3.269(1) Å, *c* = 15.296(3) Å, and β = 105.441(4)°, which is more akin to the sample discharged to 25 mAh/g but with an ∼1.3% volume increase. The crystallinity is reduced, with broader peaks observed. Therefore, with the dual-phase nature and the complexity of the unit cell, causing many of the peaks to overlap, it was difficult to carry out a reliable Rietveld refinement on the X-ray diffraction pattern. Lattice parameters were obtained from a model-independent Pawley refinement with the fit shown in Figure S4.

### 3.8 Solid-State NMR Spectroscopy

**Figure 17:**
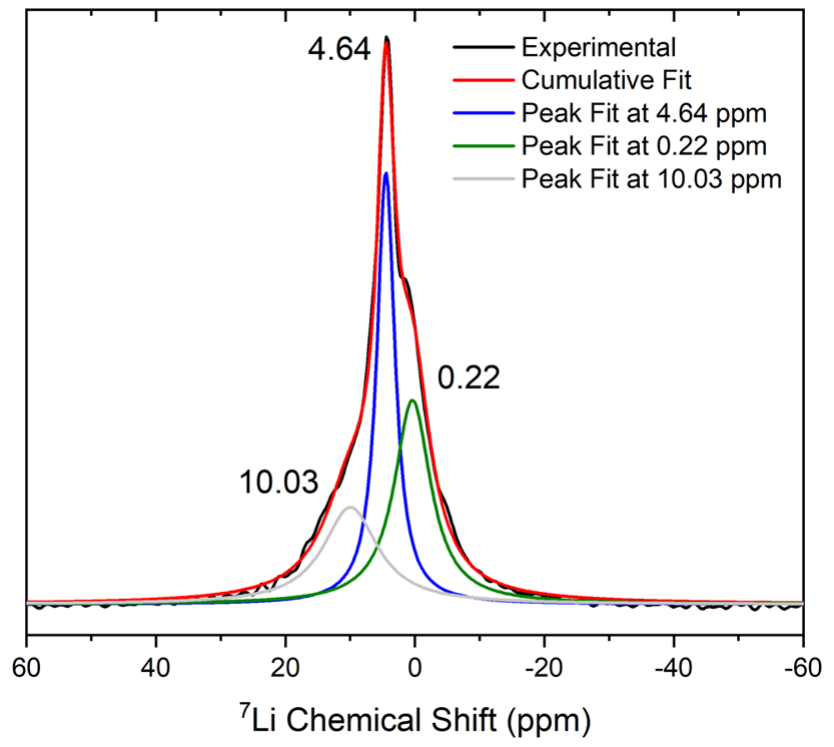
Room-temperature ^7^Li MAS NMR spectrum of Li_0.33(4)_Nb_2_Pd_0.74(1)_S_5_ showing three peaks indicating multiple Li sites on the NMR time scale.

Preliminary solid-state ^7^Li NMR spectroscopy measurements of the chemically lithiated sample Li_*x*_Nb_2_Pd_0.74_S_5_ (*x* = 0.33) produced a broad peak centered around 4 ppm, which could be deconvolved into three peaks centered around 0.22, 4.64, and 10.03 ppm (Figure 17). The peak around 0.22 ppm is assigned to diamagnetic impurities resulting from partial decomposition of the Li/NH_3_ reagent during the chemical intercalation process. The other two peaks are assigned to Li ions partially occupying the triangular prismatic sites described above and shown in Figure 9. When the electrochemically and chemically synthesized sites are compared, there is evidence from the powder pattern that the chemically synthesized sample contains a small amount of a second phase which dominates at higher Li contents (Figure 14). We deduce that in the chemically lithiated sample probed in the NMR experiment some Li ions are present in the more Li-rich second phase, leading to the appearance of the two peaks with different chemical shifts in the ^7^Li NMR spectrum. An alternative interpretation is that more than one Li site is occupied on the NMR time scale. Further NMR spectroscopy measurements across the full range of compositions and as a function of temperature would be needed to test the Li ion mobility and the distribution in these samples

## 4 Conclusions

We have shown the Li intercalated using Li/ammonia solution or intercalated electrochemically into the layered Nb_2_Pd_0.74_S_5_ superconductor led to a linear increase in the unit cell volume. The structure of the chemical intercalate, synthesized on the bulk scale, was confirmed using synchrotron X-ray and neutron powder diffraction, with the lithium being located on a triangular prismatic site with an occupancy of 0.33(4). DFT calculations provided the energies of the possible Li sites, assuming low Li occupancy, and the triangular prismatic site was the lowest in energy, in agreement with the neutron diffraction refinement.

The Li in the intercalated phase was then almost completely deintercalated using iodine, although the parent phase lattice parameters were not fully recovered, suggesting that trace amounts of Li cannot be deintercalated. Superconductivity was suppressed upon intercalation (no evidence for superconductivity above 2 K in the intercalates) but was partially restored upon deintercalation. Following a cycle of chemical Li intercalation and deintercalation, the onset transition temperature for superconductivity decreased from 6.5 K in the parent to 5.5 K in the deintercalated sample, with a significant drop in the superconducting volume fraction from 80 to 7%, possibly due to the trace Li present. The injection of electrons into the delocalized system decreased the density of states at the Fermi level and reduced the Pauli susceptibility by ∼40%, consistent with the computed density of states and the suppression of superconductivity.

Intercalation was also shown to be feasible using electrochemical methods. Nb_2_Pd_0.74_S_5_ was discharged to specific capacities of 12, 25, and 50 mAh/g, which gave theoretical Li contents of 0.19, 0.40, and 0.79, respectively, and further expansions of the unit cell which showed a volume expansion that was linear with specific capacity and hence Li content. Li_0.19_Nb_2_Pd_0.74_S_5_ is a phase-pure crystalline phase with similar structural parameters to that obtained via chemical methods. For the sample discharged to 25 mAh/g, which is more Li-rich than the chemically lithiated phase, phase separation occurred into two intercalate phases, consistent with the observation of a second phase in the chemical intercalations. Discharge to 50 mAh/g showed further changes for which further PXRD, PND, and solid-state NMR spectroscopy measurements on bulk electrochemically synthesized samples will be required for full analysis.

## Supporting Information Available

SEM-EDX data and experimental details, fitting of SQUID magnetometry curves, density of states and band structure of the intercalate, further structural parameters, and refinements (PDF)
