# Supplementary material for: Controlling the Superconductivity of Nb2PdxS5 via Reversible Li Intercalation
Source: Inorg Chem. 2024 Jan 4;63(2):1151–65. doi: 10.1021/acs.inorgchem.3c03524 (PMC10792603; doi:10.1021/acs.inorgchem.3c03524)
Supplement: Supplementary file 1 — ic3c03524_si_001.pdf [file ic3c03524_si_001.pdf]

Controlling the superconductivity of $Nb_2Pd_xS_5$ via reversible Li intercalation

Mahmoud Elgaml, [1] Sunita Dey,[2,5] Jiayi Cen,[3] Maxim Avdeev,[4,6] David O. Scanlon,[3] Clare P. Grey,[2] and Simon J. Clarke [1*]

[1]*Department of Chemistry, University of Oxford, Inorganic Chemistry Laboratory, South Parks Road, Oxford, OX1 3QR, UK.*

[2]*Department of Chemistry, University of Cambridge, Lensfield Road, Cambridge, CB2 1EW, UK*

[3]*Department of Chemistry, University College London, 20 Gordon Street, London, WC1H 0AJ, UK*

[4]*Australian Nuclear Science and Technology Organisation, New Illawarra Road, Lucas Heights, NSW 2234, Australia*

[5] *Present address: The School of Natural and Computing Sciences, University of Aberdeen, AB24 3UE, UK*

[6] *School of Chemistry, The University of Sydney, Sydney 2006, Australia*

Supporting Information

e-mail address: simon.clarke@chem.ox.ac.uk

1 SEM-EDX

Measurements were carried out on the Zeiss EVO MA10 equipped with an Oxford Instruments X-act EDX detector in the David Cockayne Centre for Electron Microscopy, University of Oxford. The powder samples were mounted on an adhesive carbon tape, and then the sample surface was coated with 6 nm carbon layers using a Lecia ACE600 Coater. The technique cannot be used for very air-sensitive samples as air exposure is inevitable during the transfer of the sample to the carbon coater and then to the sample chamber in the SEM. EDX maps were processed using the Oxford Instruments Aztec software.

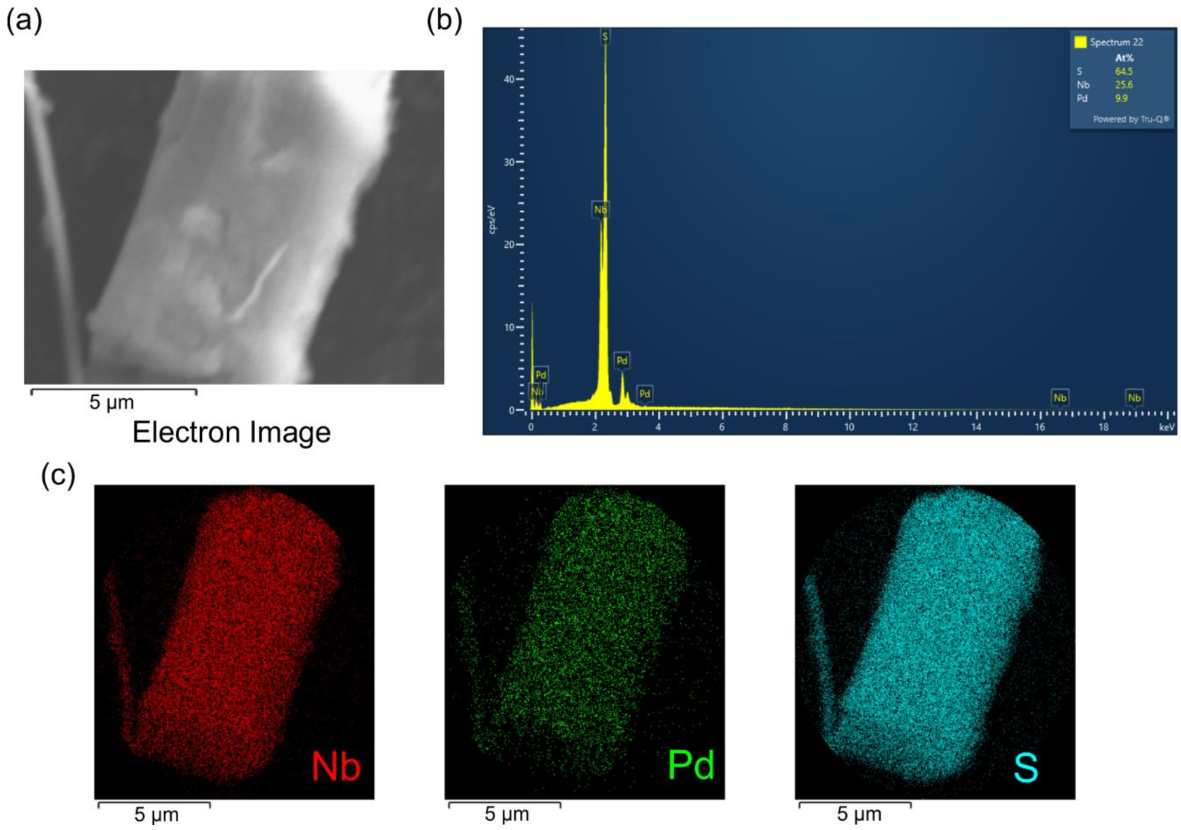

Figure S1: SEM image of $Nb_2Pd_{0.74}S_5$ with (c) showing the homogenous spread of the elements. (b) EDX spectrum giving a stoichiometry of $Nb_2Pd_{0.78(1)}S_{4.9(1)}$.

2 SQUID Magnetometry

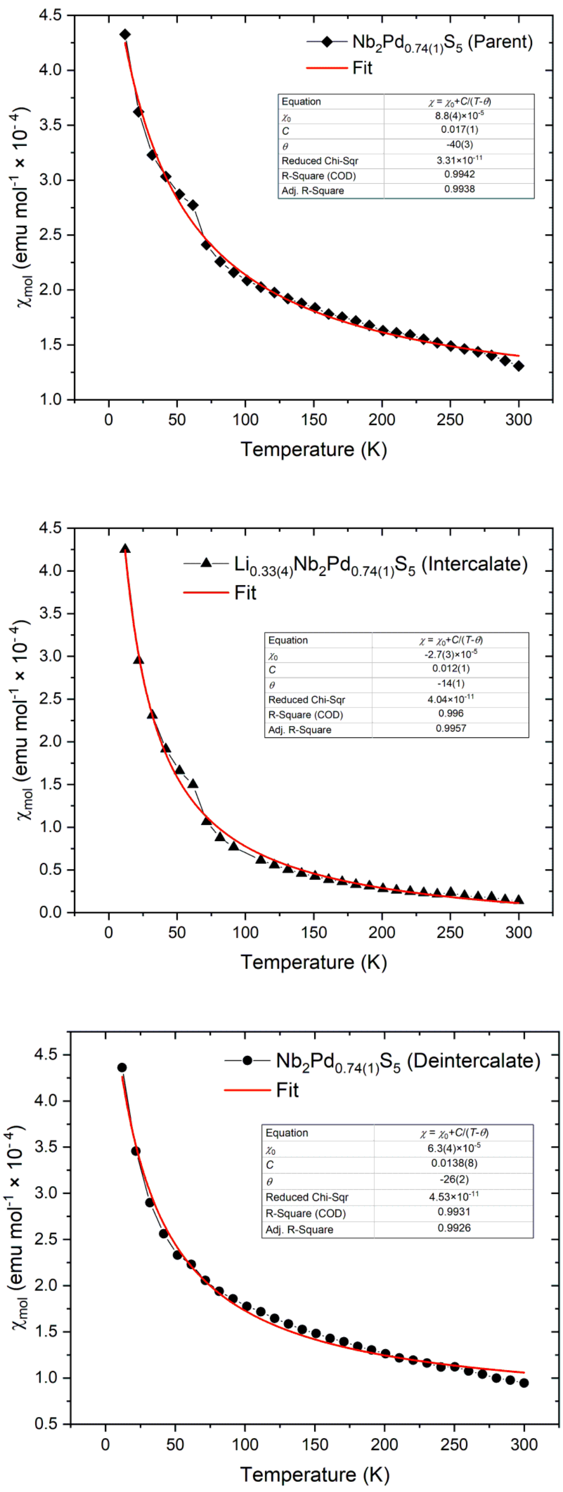

Figure S2: Molar susceptibility (χ_{mol}) against temperature of parent $Nb_2Pd_{0.74(1)}S_5$, the intercalate and deintercalated phase. The red curve shows the fit of the experimental data to the equation $\chi = \chi_0 + \frac{C}{T - \theta}$ where χ_0 is the temperature-independent susceptibility and $\frac{C}{T - \theta}$ is the Curie contribution arising from impurity or localised spin states.

3 Density of States

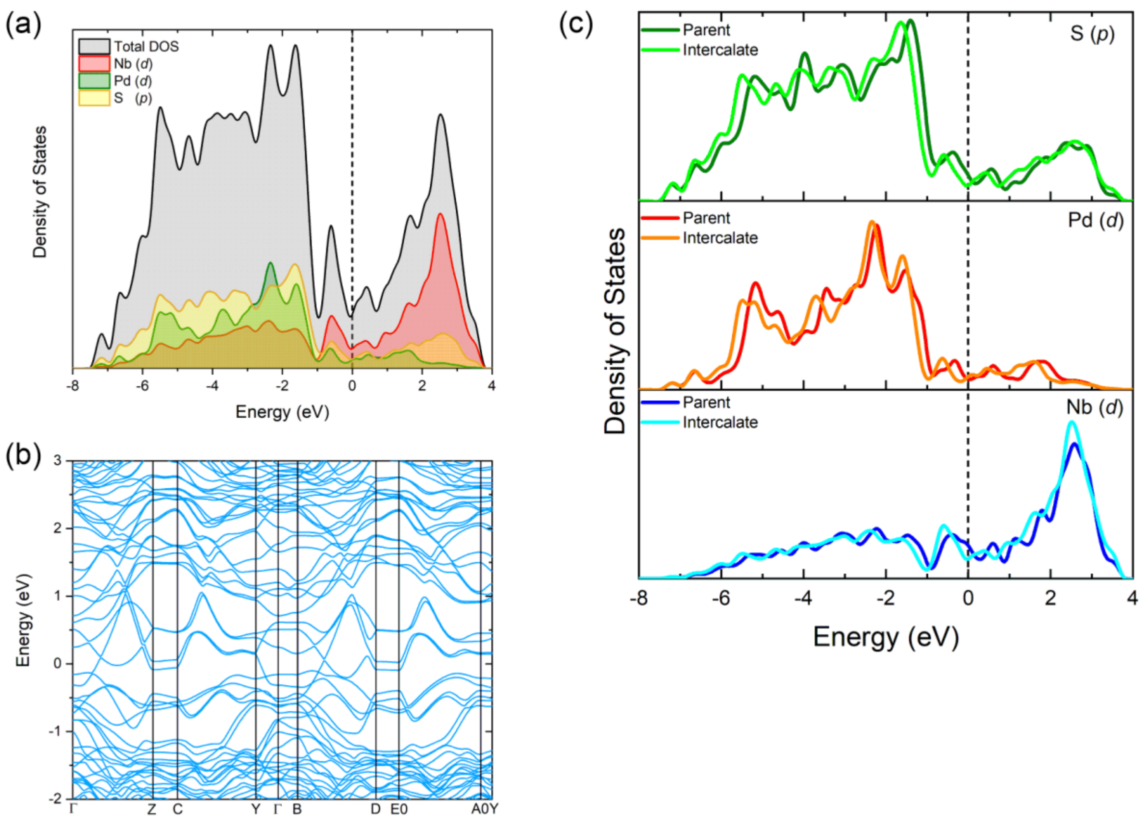

Figure S3: Electronic density of states (a) and band structure (b) for $Li_{0.25}Nb_2PdS_5$ calculated using the lowest-energy Li site. (c) shows a comparison of the partial density of states between Nb_2PdS_5 and $Li_{0.25}Nb_2PdS_5$ for the Nb (4*d*), Pd (4*d*) and S (3*p*) bands.

4 Structural Parameters

Table S1: Bond Lengths of $Nb_2Pd_{0.75}S_5$, the chemically intercalated phase, deintercalated phase and the electrochemically intercalated phases.

**Compound**	**Parent**	**Chemical Intercalate**	**Chemical Intercalate**	**Chemical Deintercalate**	**Electro-chemical Intercalate (12 mAh/g)**	**Electro-chemical Intercalate (25 mAh/g)**
**Instrument**	I11	I11	Echidna	I11	I11	I11
			**Bond Lengths / Å**			
**Nb(1)-S(2) ×1**	2.457(7)	2.464(7)	2.377(16)	2.453(7)	2.492(6)	2.432(8)
**Nb(1)-S(3) ×2**	2.472(7)	2.448(7)	2.366(14)	2.481(7)	2.452(6)	2.542(8)
**Nb(1)-S(4) ×2**	2.444(6)	2.498(6)	2.523(12)	2.449(6)	2.515(4)	2.391(6)
**Nb(1)-S(5) ×1**	2.530(9)	2.349(9)	2.444(18)	2.522(9)	2.351(8)	2.471(10)
**Nb(2)-S(1) ×1**	2.679(7)	2.724(7)	2.803(13)	2.676(7)	2.677(6)	2.649(8)
**Nb(2)-S(1) ×2**	2.530(7)	2.580(7)	2.468(13)	2.530(7)	2.550(6)	2.580(7)
**Nb(2)-S(2) ×2**	2.439(7)	2.457(7)	2.548(16)	2.437(7)	2.482(6)	2.492(8)
**Nb(2)-S(5) ×2**	2.580(5)	2.492(6)	2.430(10)	2.581(5)	2.484(5)	2.551(6)
**Pd(1)-S(3) ×2**	2.364(7)	2.313(7)	2.258(13)	2.368(7)	2.307(6)	2.304(9)
**Pd(1)-S(4) ×2**	2.325(9)	2.428(9)	2.314(13)	2.328(9)	2.446(7)	2.421(8)
**Pd(2)-S(1) ×4**	2.406(5)	2.378(6)	2.401(10)	2.408(6)	2.423(5)	2.470(6)

Table S2. Structural parameters for parent $Nb_2Pd_{0.74}S_5$.

$Nb_2Pd_{0.74(1)}S_5$ (*Z* = 4, *RMM* = 425.8(2) g mol^{-1})						
Diffractometer			I11 (PXRD)			
***Wavelength* / Å**			0.824970(5)			
***d-space range* / Å**			1.1-18.9			
***Temperature* / K**			300			
R_{wp}			3.73			
R_p			2.07			
χ^2			8.53			
Crystal System			Monoclinic			
Space Group			*C2/m* (No.12)			
***a* / Å**	***b* / Å**	***c* / Å**	***Volume* / Å^3**	***β* / °**		
12.1448(1)	3.27971(2)	15.0798(1)	585.04(1)	103.161(9)		
Positional Parameters						
Atom	***x***	***y***	***z***	***Occupancy***	***Wyckoff Parameter***	U_{iso} / Å^2
Nb(1)	0.0752(2)	0.5	0.1817(2)	1	4*i*	0.0044(6)
Nb(2)	0.1532(2)	0	0.3781(2)	1	4*i*	0.0044(6)
Pd(1)	0	0	0	1	2*a*	0.0022(9)
Pd(2)	0	0	0.5	0.496(3)	2*c*	0.0022(9)
S(1)	0.3538(5)	0	0.4917(5)	1	4*i*	0.0043(7)
S(2)	0.2511(5)	0.5	0.3032(5)	1	4*i*	0.0043(7)
S(3)	0.1789(5)	0	0.1032(5)	1	4*i*	0.0043(7)
S(4)	0.4276(5)	0.5	0.1319(5)	1	4*i*	0.0043(7)
S(5)	0.4995(4)	0	0.3191(4)	1	4*i*	0.0043(7)

Table S3. Structural parameters of the deintercalated compound.

**$Nb_2Pd_{0.74(1)}S_5$ (*Z* = 4, *RMM* = 424.8(2) g mol^{-1})**						
Diffractometer			I11 (PXRD)			
***Wavelength* / Å**			0.824970(5)			
***d-space Range* / Å**			1.1-18.9			
***Temperature* / K**			300			
R_{wp}			3.14			
R_p			2.54			
χ^2			8.87			
Crystal System			Monoclinic			
Space Group			*C2/m* (No.12)			
***a* / Å**	***b* / Å**	***c* / Å**	***Volume* / Å^3**	***β* / °**		
12.1892(2)	3.28279(3)	15.1410(2)	588.10(2)	103.906(2)		
Positional Parameters						
Atom	***x***	***y***	***z***	***Occupancy***	***Wyckoff Parameter***	**U_{iso} / Å^2**
Nb(1)	0.0758(2)	0.5	0.1817(2)	1	4*i*	0.0012(5)
Nb(2)	0.1512(2)	0	0.3781(2)	1	4*i*	0.0012(5)
Pd(1)	0	0	0	1	2*a*	0.0028(8)
Pd(2)	0	0	0.5	0.479(3)	2*c*	0.0028(8)
S(1)	0.3542(6)	0	0.4956(5)	1	4*i*	0.0016(6)
S(2)	0.2488(6)	0.5	0.3060(5)	1	4*i*	0.0016(6)
S(3)	0.1763(6)	0	0.1082(5)	1	4*i*	0.0016(6)
S(4)	0.4259(6)	0.5	0.1281(5)	1	4*i*	0.0016(6)
S(5)	0.4870(5)	0	0.3175(5)	1	4*i*	0.0016(6)

Table S4. Structural Parameters of the electrochemical sample discharge to 12 mAh/g (x(Li) = 0.19).

**$Li_xNb_2Pd_{0.75(1)}S_5$ (*Z* = 4, *RMM* = 427.3(1) g mol^{-1})** [a]						
Diffractometer			I11 (PXRD)			
***Wavelength* / Å**			0.824970(5)			
***d-space Range* / Å**			1.1-18.9			
***Temperature* / K**			300			
**R_{wp}**			1.23			
**R_p**			2.11			
**χ^2**			3.27			
Crystal System			Monoclinic			
Space Group			*C2/m* (No.12)			
***a* / Å**	***b* / Å**	***c* / Å**	***Volume* / Å^3**	***β* / °**		
12.3266(3)	3.29196(6)	15.3412(3)	597.32(2)	106.360(1)		
Positional Parameters						
Atom	***x***	***y***	***z***	***Occupancy***	***Wyckoff Parameter***	**U_{iso} / Å^2**
Nb(1)	0.07370(18)	0.5	0.18443(13)	1	4*i*	0.0023(6)
Nb(2)	0.14760(17)	0	0.37780(13)	1	4*i*	0.0031(6)
Pd(1)	0	0	0	1	2*a*	0.0015(6)
Pd(2)	0	0	0.5	0.501(2)	2*c*	0.0015(6)
S(1)	0.3543(5)	0	0.4957(4)	1	4*i*	0.0049(11)
S(2)	0.2522(5)	0.5	0.3111(4)	1	4*i*	0.0049(11)
S(3)	0.1691(5)	0	0.1132(4)	1	4*i*	0.0016(15)
S(4)	0.4155(4)	0.5	0.1264(4)	1	4*i*	0.0096(18)
S(5)	0.4971(5)	0	0.3085(4)	1	4*i*	0.0077(17)

[a] Note that the RMM is an estimate due to the unknown value of the Li occupancy. The RMM was calculated based off x(Li) = 0.19 used in the reaction.

Table S5. Structural Parameters of the electrochemical sample discharge to 25 mAh/g (x(Li) = 0.40).

**$Li_xNb_2Pd_{0.75(2)}S_5$ (Z = 4, RMM = 429.7(2) g mol^{-1}) [a]**						
Diffractometer			I11 (PXRD)			
***Wavelength* / Å**			0.824970(5)			
***d-space Range* / Å**			1.1-18.9			
***Temperature* / K**			300			
**R_{wp}**			1.00			
**R_p**			0.70			
**χ^2**			2.65			
Crystal System			Monoclinic			
Space Group			*C2/m* (No.12)			
***a* / Å**	***b* / Å**	***c* / Å**	***Volume* / Å^3**	***β* / °**		
12.6208(4)	3.30577(8)	15.2050(4)	611.61(3)	105.396(1)		
Positional Parameters						
Atom	***x***	***y***	***z***	***Occupancy***	***Wyckoff Parameter***	**U_{iso} / Å^2**
Nb(1)	0.0744(3)	0.5	0.1851(2)	1	4*i*	0.0116(9)
Nb(2)	0.1534(3)	0	0.3784(2)	1	4*i*	0.0074(8)
Pd(1)	0	0	0	1	2*a*	0.0035(8)
Pd(2)	0	0	0.5	0.518(3)	2*c*	0.0035(8)
S(1)	0.3525(6)	0	0.4927(5)	1	4*i*	0.0013(7)
S(2)	0.2479(7)	0.5	0.3027(5)	1	4*i*	0.0013(7)
S(3)	0.1774(7)	0	0.1112(5)	1	4*i*	0.0013(7)
S(4)	0.4362(6)	0.5	0.1286(5)	1	4*i*	0.0013(7)
S(5)	0.4970(6)	0	0.3183(4)	1	4*i*	0.0013(7)

[a] Note that the RMM is an estimate due to the unknown value of the Li occupancy. The RMM was calculated based off x(Li) = 0.40 used in the reaction.

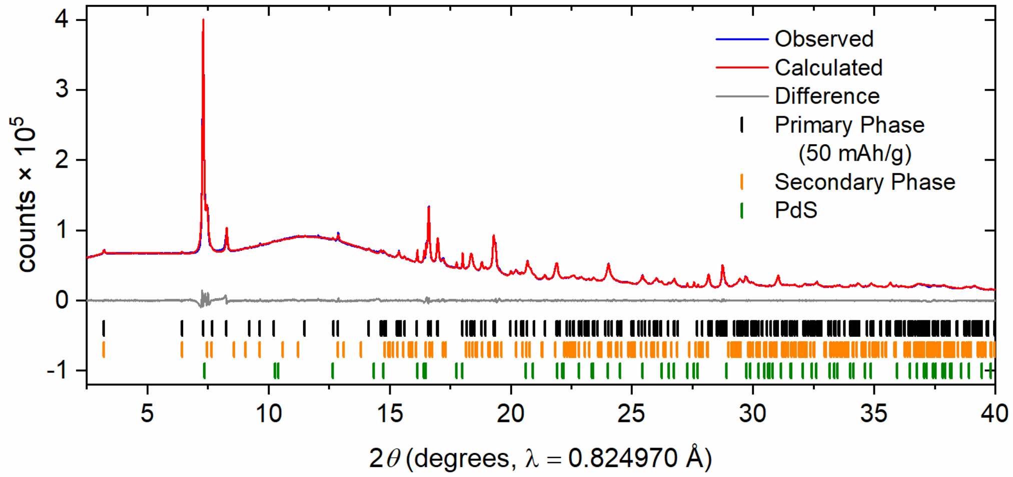

Figure S4. Pawley refinement for the sample discharged to 50 mAh/g (x(Li)=0.8). R_{wp}: 1.20 %, R_p: 0.72 %, χ^2: 3.19 %.

5 Note for Tables.

In the Rietveld refinement, the function s_y is minimised

$$S_y = \sum_i w_i (y_i - y_{ci})^2$$

where y_i is the observed, and y_{ci} is the calculated intensity at point i and w_i is the weighting factor, defined by $\frac{1}{y_i}$

The weighted profile R factor, R_{wp}, is

$$R_{wp} = \sqrt{\frac{\sum_i w_i (y_i - y_{ci})^2}{\sum_i w_i y_i^2}}$$

The profile R factor, R_p is

$$R_p = \sqrt{\frac{\sum_i |y_i - y_{ci}|}{\sum_i y_i}}$$

The statistically expected R value, R_{exp}, in which all deviations of the calculated pattern from the observed are due to statistical variations. R_{exp} is defined by:

$$R_{exp} = \sqrt{\frac{N_{obs} - N_{var}}{\sum_i w_i y_i^2}}$$

where N_{obs} and N_{var} are the number of observables and number of variables, respectively.

A goodness of fit parameter, χ^2, is defined from the square of the ratio of R_{wp} and $R_{exp.}$
